# Macrolides from *Streptomyces* sp. SN5452 and Their Antifungal Activity against *Pyricularia oryzae*

**DOI:** 10.3390/microorganisms10081612

**Published:** 2022-08-09

**Authors:** Yinan Wang, Di Yang, Yuhui Bi, Zhiguo Yu

**Affiliations:** 1 College of Plant Protection, Shenyang Agricultural University, Shenyang 110866, China; 2 Engineering & Technological Research Center of Biopesticide for Liaoning Province, Shenyang 110866, China

**Keywords:** *Streptomyces*, macrolides, venturicidins, *Pyricularia oryzae*, antifungal activity

## Abstract

*Pyricularia oryzae* causes rice blast, the major destructive disease in nearly all rice fields. In order to obtain highly active compounds against *P. oryzae*, four new 20-membered macrolides named venturicidins G–J (**1–4**) were isolated from the culture broth of *Streptomyces* sp. SN5452 along with two known ones, venturicidins A (**5**) and B (**6**). Their structures were determined by the cumulative analyses of nuclear magnetic resonance (NMR) spectroscopy and high-resolution electrospray ionization mass spectrometry (HRESIMS) data. All isolated compounds were evaluated for their antifungal activity against *P. oryzae*. Interestingly, these compounds exhibited obvious inhibition to mycelial growth and conidial germination of *P. oryzae*. Remarkably, the EC_50_ values of venturicidins A (**5**), B (**6**), and I (**3**) against mycelial growth were 0.11, 0.15 and 0.35 µg/mL, and their EC_50_ values of conidial germination were 0.27, 0.39 and 1.14 µg/mL, respectively. The analysis of structure-activity relationships (SARs) revealed that the methylated positions might be involved in the antifungal activity of venturicidins. These results indicate that the venturicidins are prospective candidates for novel fungicides that can be applied in controlling rice blast.

## 1. Introduction

The need for food quality and quantity is urgent due to the increase in population and the improvement of people’s living standards [1]. Rice is the staple food for most peoples in different countries around the world [2], including China, Bangladesh, and Malaysia. However, the yield of rice is threatened by a variety of plant diseases every year, among which rice blast can cause up to 30% yield loss in some regions [3,4]. Rice blast is caused by *Pyricularia oryzae* [5], a haploid filamentous ascomycete [6,7], which can infect any stage of plant growth, causing leaf blast, node blast and panicle blast [8,9,10,11].

At present, the control of rice blast mainly depends on applying synthetic chemical agents [12,13]. These have been repeatedly applied for decades, which not only develops resistance of *P. oryzae* to them [14,15], but also destroys the balance of ecosystem [16]. Moreover, after several years of planting disease-resistant varieties [17], the pathogens may develop new pathogenic races, resulting in the collapse of resistance [11,18]. In order to solve these difficulties, scientists expect to seek for environmentally friendly and safe microbial biocontrol agents.

Actinomycetes, renowned for their ability to produce various novel bioactive compounds, were applied in the agricultural field as microbial biocontrol agents [19,20,21]. *Streptomyces* is the largest genus of the phylum *Actinobacteria*, which produces abundant secondary metabolites such as macrolides, terpenoids, alkaloids, flavones, and polyketides [22,23]. In previous studies, these secondary metabolites were considered as crop protection agents, due to their antimicrobial, insecticidal and herbicidal activities [24,25,26,27].

In our ongoing efforts to seek for new and bioactive secondary metabolites produced by actinomycetes [28,29], the crude extract from the fermentation culture of *Streptomyces* sp. SN5452 at 50 μg/mL could completely inhibit the mycelial growth of *P. oryzae* (Figure S1). In this study, we isolated and purified the active compounds from the crude extract. The structures of these active compounds were determined based on the analysis of mass spectrometry (MS) and nuclear magnetic resonance (NMR) data. Furthermore, their antifungal activity against *P. oryzae* was also evaluated with mycelial growth inhibition and conidial germination assays.

## 2. Materials and Methods

### 2.1. General Experimental Procedures

Optical rotations were obtained at the sodium D line with a polarimeter (Atago, Tokyo, Japan), maintained at room temperature. NMR spectra experiments were operated on an Avance-600 NMR spectrometer (Bruker, Karlsruhe, Germany). Chemical shifts were calibrated by carbon signals and the residual proton signals of DMSO-*d_6_* (*δ*_C_ 39.5 and *δ*_H_ 2.50). High-resolution mass spectra (HRESIMS) were recorded on an Agilent 1260/6520 Q-TOF mass spectrometer. The crude extract was chromatographed on silica column with silica gel of 100–200 and 200–300 mesh (Qingdao Ocean Chemical Co., Ltd., Qingdao, China) and Sephadex LH-20 (GE Healthcare, Uppsala, Sweden). High-performance liquid chromatography (HPLC) analysis was performed using the C18 column (Agilent ZORBAX Eclipse XDB, 4.60 × 250 mm, 5 μm) on an Agilent 1260 series system (Agilent, Santa Clara, CA, USA). Active compounds were collected using semi-preparative HPLC with a C18 column (Agilent ZORBAX Eclipse XDB, 9.4 × 250 mm, 5 μm). The germination number of conidia was observed by microscope (Nikon, Tokyo, Japan). All chemical agents were purchased from Sinopharm Chemical Reagent company (Shanghai, China).

### 2.2. Actinomycete Material

The stain was isolated from the gut of millipede (*Kronopolites svenhedind* Verhoeff) which was obtained from campus of Shenyang Agricultural University. The processing of the Diplopoda gut sample and the isolation of the strain were conducted according to Heo et al.’s method [30]. For taxonomic identification, the strain 16S rRNA sequence was compared and analyzed by EzTaxon database. A phylogenetic tree was constructed based on 16S rRNA sequence using Molecular Evolutionary Genetics (MEGA v 7.0) [31,32]. Colonies were deposited in 20% (*v/v*) glycerol solution at −80 °C.

### 2.3. Fermentation and Extraction

*Streptomyces* sp. SN5452 was cultured on Gause’s synthetic agar no. 1 (GS) [33] plates at 28 °C in the dark for 10 days for subsequent fermentation. The mycelia were inoculated into test tubes with 5 mL liquid ISP 2 medium [34] and shaken (180 rpm) at 28 °C in the dark for 2 days, and then were transferred to 250 mL Erlenmeyer flask with 50 mL liquid ISP 2 medium with shaking for 2 days to prepare the seed culture. Finally, the seed culture was shifted into 2 L Erlenmeyer flasks, which contained 400 mL of the GS liquid medium and 16 g of Amberlite XAD-16 resin. A total of 48 L fermentation culture was obtained after cultivating for 7 days under identical conditions.

The resin was collected from the fermentation broth by repeated deionized water washing. After it was dried in an oven at 30 °C to remove moisture, the resin was extracted four times with CH_3_OH. The CH_3_OH fractions were pooled and concentrated to obtain the CH_3_OH extract, and then it was redissolved in 50% CH_3_OH in H_2_O (0.6 L). The solution was extracted four times with the same volume of CH_2_Cl_2_. Collecting CH_2_Cl_2_ fractions were concentrated to produce 11 g of crude extract.

### 2.4. Isolation and Purification

The purification of concentrated crude extract was operated by normal phase silica gel chromatography (6 cm × 40 cm), using a gradient of CH_3_OH-CH_2_Cl_2_ solvent (100:0, 50:1, 25:1, 25:2, 25:4, 1:1 and 0:100, 2 L each) as the mobile phase, which produced three fractions, A–C. Fraction B was further fractionated via silica gel chromatography with petroleum ether-ethyl acetate (7:3, 6.5:3.5, 6:4, 5.5:4.5 and 1:1, 1 L each) as the mobile phase to obtain B_1_ and B_2_ fractions. Fraction B_1_ was purified using Sephadex LH-20 column with petroleum ether-dichloromethane-methanol (2:1:1) as eluent to remove impurities. Then, it was isolated by reverse-phase semipreparative HPLC, and eluted with 80% CH_3_OH in H_2_O to yield compounds **1** (3.2 mg, *t*_R_ = 18.04 min), **6** (71.5 mg, *t*_R_ = 19.86 min), and **2** (8.1 mg, *t*_R_ = 31.62 min). Fraction B_2_ was also separated applying reverse-phase semipreparative HPLC with the same chromatographic separation conditions as fraction B_1_, yielding compounds **5** (101.6 mg), **3** (4.5 mg) and **4** (10.3 mg) at 17.57, 20.57 and 26.36 min, respectively.

Venturicidin G, **1**. White power; ${\left[\alpha \right]}_{D}^{24}+33.33$ *c* 0.3, CH_3_OH); HRESIMS *m/z* 715.4400 [M + Na]^+^ (calcd for C_39_H_64_O_10_Na, 715.4397).

Venturicidin H, **2**. White power; ${\left[\alpha \right]}_{D}^{24}+50.00$ (*c* 0.40, CH_3_OH); HRESIMS *m/z* 743.4695 [M + Na]^+^ (calcd for C_41_H_68_O_10_Na, 743.4710).

Venturicidin I, **3**. White power; ${\left[\alpha \right]}_{D}^{24}+48.78$ (*c* 0.41, CH_3_OH); HRESIMS *m/z* 753.4900 [M + NH_4_]^+^ (calcd for C_40_H_69_N_2_O_11_, 753.4943).

Venturicidin J, **4**. White power; ${\left[\alpha \right]}_{D}^{24}+50.00$ (*c* 0.40, CH_3_OH); HRESIMS *m/z* 781.5243 [M + NH_4_]^+^ (calcd for C_42_H_73_N_2_O_11_, 781.5256).

Venturicidin G–J (**1–4**), ^1^H and ^13^C NMR data (*d*_6_-DMSO), see Table 1 and Table 2.

### 2.5. Effect of Compounds on the Mycelial Growth of P. oryzae

Mycelial growth inhibition assay was performed as described by Li et al. [35] to measure the inhibitory effect of compounds **1–6** on *P. oryzae*. The tested compounds were dissolved in sterile water containing 0.5% DMSO and 0.25% Tween-80 to produce a desired agent concentration of 0.078125–8 µg/mL. The agents were blended with 40–45 °C potato dextrose agar, in which 0 µg/mL represented the negative control. The carbendazim was considered as the positive control. The 5 mm plugs of *P. oryzae* were placed in the center of treated plates (d = 90 mm). After 15 days of dark culture at 25 °C, the colony diameter of each test group was measured. Experiments were repeated in three replicates. The antifungal activities were calculated using the following formula:

inhibition (%) = (D_C_ − D_T_)/(D_C_ − 5) × 100

among which, D_C_ is colony diameter in the negative control plate and D_T_ is colony diameter in the plate containing tested compounds.

### 2.6. Effect of Compounds on the Conidia Germination of P. oryzae

*P. oryzae* was incubated on oatmeal tomato agar medium as described by Miao et al. [36]. When *P. oryzae* reached the edge of the plate, the aerial growth was scraped off with sterilized cotton swabs. They were then placed in an incubator with black light lamp at 25 °C. After 7 days, the conidia were harvested from conidial colonies. The concentrations of conidia in suspension were adjusted to 1 × 10^5^ conidia per mL by a hemocytometer [37] and 30 μL of various concentrations (0.078125–100 µg/mL) of tested compounds were dropped onto the glass slides with 30 μL conidial suspension before incubating both of them for 6 h. The sterile water containing 0.5% DMSO and 0.25% Tween-80 was considered as the negative control. The carbendazim was considered as the positive control. The conidia were considered to have germinated when the length of the germ tube was greater than the short radius of the conidia. When the negative control conidial germination rate was greater than 80%, the number of conidial germinated at various concentration was observed and assessed by under the microscope. Experiments were repeated in three replicates.

## 3. Results

### 3.1. Identification of Strain SN5452

The 16S rRNA gene sequence analysis and comparisons showed that strain SN5452 was affiliated with the genus *Streptomyces* and shared the greatest gene sequence similarity to *Streptomyces setonii* (99.79%). The phylogenetic analysis of the 16S rRNA gene sequences indicated the stain forms a cluster with *Streptomyces clavifer* NRRL B-2557^T^, *Streptomyces mutomycini* NRRL B-65393^T^, *Streptomyces atroolivaceus* NRRL ISP-5137^T^ and *Streptomyces finlayi* NRRL B-12114^T^ (Figure S2). Therefore, the strain SN5452 belongs to the genus *Streptomyces* and was named *Streptomyces* sp. SN5452 (Genbank accession no. ON358333).

### 3.2. Extraction, Separation and Purifcation of Extract

**Figure 1:**
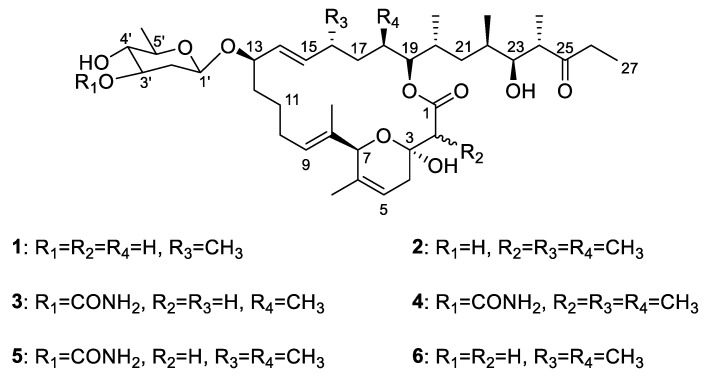
Structures of compounds **1–6**.

The CH_2_Cl_2_ extract from the fermentation culture of *Streptomyces* sp. SN5452 was chromatographed on silica gel column followed by further purification on Sephadex LH-20 columns and reversed-phase HPLC, to afford compounds **1–6**. Analysis of ESI-MS data and ^1^H and ^13^C NMR spectra suggested **5** and **6** to be venturicidins A and B, respectively, whose identities were unambiguously confirmed by extensive 2D NMR (COSY, HSQC and gHMBC) spectroscopic analyses as well as comparison to previously reported spectroscopic data (Figure 1) [38].

### 3.3. Structure Elucidation of Compounds

**Figure 2:**
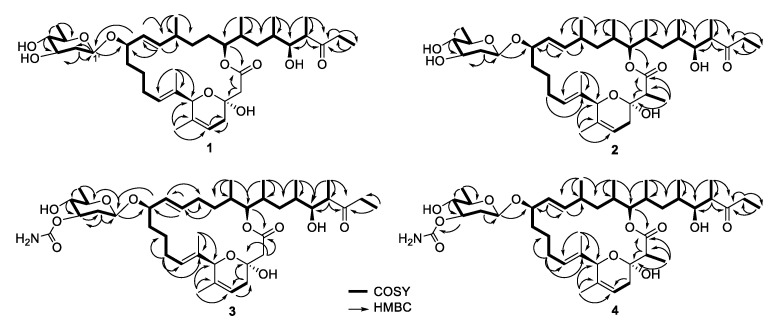
2D NMR correlations of compounds **1**–**4**. 2D NMR: two-dimensional nuclear magnetic resonance.

Compound **1** was obtained as a white powder. Its molecular formula, C_39_H_64_O_10_, was established on the basis of HRESIMS data, indicating eight degrees of unsaturation (Figure S8). The ^1^H NMR spectrum of **1** showed eight methyl proton signals at *δ*_H_ 0.71–1.41 and four olefinic proton signals [*δ*_H_ 5.15 (1H, dd), 5.30 (1H, dd), 5.38 (1H, m), 5.45 (1H, m)] (Table 1 and Figure S3). The ^13^C NMR and HSQC spectra of **1** showed eight methyl groups, ten methylene groups, sixteen methine groups, and five quaternary carbons (Figures S4 and S5). The spectroscopic data also revealed two olefinic, one ketone, one ketal and one lactone carbon signals. These results indicated that **1** possesses the same venturicidin scaffold as that of **6** (Figure 2). Analyses of the ^1^H and ^13^C NMR spectra of **1** and **6** showed that one methyl group (*δ*_H_ 0.78; *δ*_C_ 12.9) and one methine group (*δ*_H_ 1.80; *δ*_C_ 34.6) found in **6** were not present in **1** (Table S1). Instead, **1** contains an additional methylene group (*δ*_H_ 1.29, 1.47; *δ*_C_ 29.0). The HMBC cross peaks of H_2_-18 (*δ*_H_ 1.29, 1.47) with C-17 (*δ*_C_ 32.8) and C-19 (*δ*_C_ 78.3) indicated the methylene is located at C-18 (Figure 2 and Figure S6). Therefore, the structure of **1** differs from that of **6** in only one aspect: the methyl group at C-18 in **6** is replaced by a proton in **1**. This was also confirmed by the HRESIMS data and molecular formulas of **1** and **6** which indicated **6** has one more carbon and two more hydrogens than **1**. The structure of compound **1** was thus elucidated, and compound **1** was named as venturicidin G.

Compound **2** was purified as a white powder. The molecular formula of **2** was determined as C_41_H_68_O_10_ based on HRESIMS data (Figure S15). Similarly, **2** was identified to have the same scaffold as **6** based on their spectroscopic data (Figures S9–S14). However, the ^1^H and ^13^C NMR data revealed that the signals of methylene (*δ*_H_ 2.73, 2.54; *δ*_C_ 43.7) in **6** were substituted by signals of a methyl group (*δ*_H_ 1.12; *δ*_C_ 13.0) and a methine group (*δ*_H_ 2.57; *δ*_C_ 47.6) in **2**, respectively (Table 1, Table 2 and Table S1). The HMBC correlations of H_3_-2CH_3_ (*δ*_H_ 1.12) with C-1 (*δ*_C_ 176.0), C-2 (*δ*_C_ 47.6) and C-3 (*δ*_C_ 95.7) and COSY correlation between H_3_-2CH_3_ (*δ*_H_ 1.12) and H-2 (*δ*_H_ 2.57) illustrated the position of the methyl at C-2 (Figure 2). From these results, the structure of compound **2** was elucidated, and compound **2** was named as venturicidin H.

Compound **3** was obtained as white powder. Its molecular formula, C_40_H_65_NO_11_, was inferred by HRESIMS data (Figure S21). The NMR data exhibited structure of **3** was highly similar to that of **5** (Figure S16–S20). The analysis of ^1^H NMR data of **5** exhibited a methyl (H_3_-16CH_3_, *δ*_H_ 0.89) that was absent in **3**, while **3** had a methylene at H_2_-16 (*δ*_H_ 2.04, 1.97) rather than a methine at H-16 (*δ*_H_ 2.06) in **5** (Table 1 and Table S1). The HMBC correlations of H_2_-16 with C-15 (*δ*_C_ 137.4) and C-17 (*δ*_C_ 34.3) also revealed the methyl (*δ*_H_ 0.89) in **5** was missing in **3** (Figure 2), corresponding to a 14 Da mass decrease. Consequently, **3** was elucidated and named as venturicidin I.

Compound **4** was isolated as white powder, and revealed spectroscopic data remarkable similar with those of **5** (Figure S22–S27). The molecular formula of **4** was determined as C_42_H_69_NO_11_ compatibly with its HRESIMS data (Figure S28), which was 14 amu higher than that of **5**, suggesting the presence of an additional methyl group (*δ*_H_ 1.13, *δ*_C_ 13.0) in **4** (Table 1 and Table 2). The HMBC correlations of H_3_-2CH_3_ (*δ*_H_ 1.13) with C-1 (*δ*_C_ 176.0), C-2 (*δ*_C_ 47.6) and C-3 (*δ*_C_ 95.7) and COSY correlation between H_3_-2CH_3_ (*δ*_H_ 1.13) and H-2 (*δ*_H_ 2.57) confirmed that the additional methyl was located at C-2 of **4** (Figure 2). Therefore, the structure of **4** was determined as illustrated in Figure 1, and compound **4** was named as venturicidin J.

### 3.4. Antifungal Activity Assay

**Figure 3:**
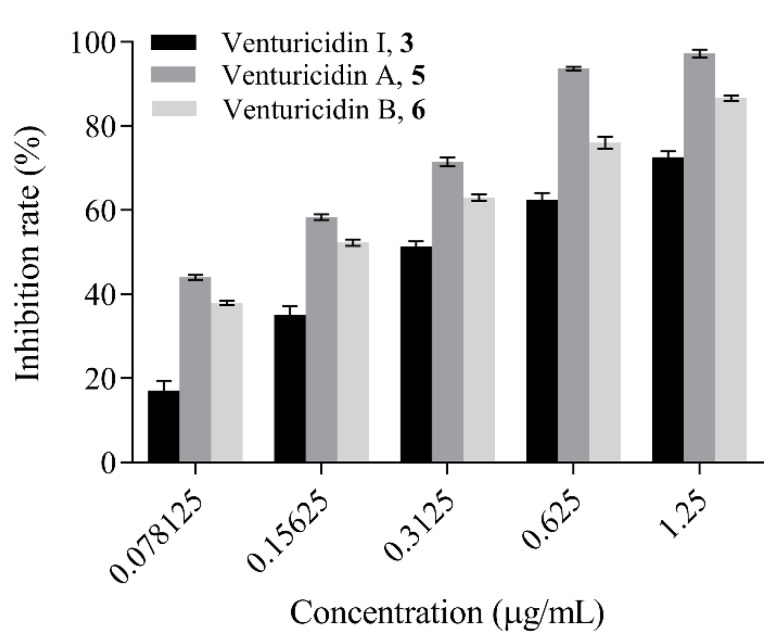
Effect of venturicidin I, **3**, venturicidin A, **5**, and venturicidin B, **6** on mycelial growth of *P. oryzae*.

Compounds **1–6** were evaluated for their antifungal activities against *P. oryzae* in mycelial growth inhibition and conidial germination assays. The results showed EC_50_ values of the compounds **1–6** against mycelial growth were approximately 1.78, 1.43, 0.35, 1.40, 0.11, 0.15 µg/mL, respectively (Table 3). Notably, the compounds **3**, **5** and **6** remarkably limit the mycelial radial elongation of *P. oryzae* (Figure 3), which showed comparable antifungal activity to the positive control carbendazim (EC_50_ = 0.30 µg/mL). Compounds **1**, **2** and **4** showed moderate antifungal activities.

In addition to inhibiting mycelial growth, compounds **1–6** also inhibited the conidial germination of *P. oryzae* in different levels, with the EC_50_ values estimated to be 24.95, 5.55, 1.14, 4.49, 0.27 and 0.39 µg/mL, respectively (Table 3). These results indicated that compounds **5** and **6** exert higher antimicrobial activity against *P. oryzae* than the positive control carbendazim (EC_50_ = 3.99 µg/mL). Compounds **2**, **3** and **4** showed comparable antifungal activity to the positive control, and compound **1** exhibited weaker inhibitory activity than carbendazim.

### 3.5. Structure Activity Relationship Analysis

The relationship between the structures and antifungal activity was elucidated based on the compounds’ molecular structures, and the discussion upon their antimicrobial activity results against different developmental stages of *P. oryzae*. Both compounds **5** and **6** showed similar antifungal activity against *P. oryzae*, indicating the presence of acylamino group at the C-3 position does not cause a prominent effect on its activity. Comparing the structures of **1** with **6**, a methyl group at the C-18 (R_4_) was replaced by a hydrogen atom in **1**, which impairs the antifungal activity against *P. oryzae*, so they have large activity differences in conidial germination inhibition. In contrast, between **3** and **5** the replacement in **3** happened on C-16 (R_3_), which indicates that they have no differences in antifungal activity. Thus, the methyl group in position R_4_ seems to increase the activity. Both compounds **2** and **4** introduce a methyl group at the C-2 position, lower the antimicrobial activity in comparison with that of **5** and **6**. These results suggested that the antifungal activities of these compounds are influenced by the methylated positions in venturicidins. The SARs of these compounds provided a scientific basis for discovery of potent fungicides.

## 4. Discussion

With the increasing devastating rice blast disease, *P. oryzae* has provoked severe loss in the world [39]. The control of rice blast remains a long-standing agricultural issue associated with highly variable nature of *P. oryzae* [37,40]. In addition, *P. oryzae* has developed resistance to most of commercially available fungicides [16]. Thus, novel fungicides are a constant and critical need. Recently, microbial pesticides are widely applied in the control of plant diseases due to friendliness to the environment [41]. Unfortunately, there are few reports on the microbial pesticides against *P. oryzae*. This study indicated that secondary metabolites of *Streptomyces* sp. SN5452 may be promising fungicides in the control of rice blast, because they strongly inhibit the mycelial growth and the conidial germination of *P. oryzae*. The isolation and screening of *Streptomyces* are the prerequisites for obtaining bioactive natural products [42]. In our study, *Streptomyces* sp. SN5452 was isolated and purified from the gut of a millipede. By the analysis of 16S rRNA gene sequence, the strain belonged to *Streptomyces* genus, and the crude extract made from the fermentation culture of the strain showed prominent inhibitory activity against *P. oryzae*.

*Streptomyces* produce a number of secondary metabolites used in fields. The commercial kasugamycin produced by *Streptomyces kasugaensis* showed potential preventive effects against rice blast [43]. The insecticide avermectin has been used extensively for controlling *Plutella xylostella* and *Pieris rapae* in fields, which was produced by *Streptomyces avermitilis* [44,45,46]. Furthermore, *Streptomyces* fermentation products are considered as a valuable resource for the development of novel pesticides. The dimethyl sulfide and trimethyl sulfide produced by *Streptomyces* sp. AN090126, showed broad-spectrum antimicrobial activity against various plant-pathogenic bacteria and fungi, including *Ralstonia solanacearum*, *Xanthomonas euvesicatoria*, *Sclerotinia homoeocarpa* [47]. The LC_50_ value of endostemonine J, an ionophore antibiotic produced by *Streptomyces* sp. BS-1, against *Aphis gossypii* was 3.55 μg/mL at 72 h via leaf dipping assay [48]. Compound cinnoline-4-carboxylic acid was isolated from the fermentation culture of *Streptomyces* sp. KRA17-580 by Kim’s group, and completely inhibited the growth *Digitaria ciliaris* at a concentration of 50 μg/mL [49]. In this study, venturicidins A, B and venturicidins G-J were isolated from the fermentation culture of *Streptomyces* sp. SN5452. These compounds inhibited mycelial growth and conidial germination of *P. oryzae*, with EC_50_ values ranging from 0.11 to 1.78 μg/mL and from 0.27 to 24.95 μg/mL, respectively.

As we all know, structures of compounds were determined by the cumulative analyses of NMR, MS and X-ray data. The current study showed that the absolute configuration of compounds **1**, **3**, **5** and **6** were elucidated based on NMR and MS analyses and previously reported X-ray data [46]. However, the absolute configuration of compounds **2** and **4** were uncertain by spectroscopic data. We also tried to cultivate single-crystals of the compounds **2** and **4**, but unfortunately it was not successful due to their structural specificity. Named as venturicidins G-J, compounds **1–4** enriched the structural diversity of 20-membered macrolide compounds [50,51,52,53,54,55], and compounds **5–6** were identified as venturicidins A–B. Further, venturicidins A, B and I exhibited good inhibition to mycelial growth and conidial germination of *P. oryzae*, which was comparable or superior to the positive control carbendazim. However, the actual field efficacy of these compounds should be further studied.

## 5. Conclusions

In conclusion, the crude extract made from the fermentation culture of *Streptomyces* sp. SN5452 showed inhibitory activity against *P. oryzae*. Compounds **1**, **2**, **3** and **4** were isolated and identified for the first time from the fermentation culture of *Streptomyces* sp. SN5452. Compounds **3**, **5** and **6** exhibited good antimicrobial activity against *P. oryzae*. Given that microbial secondary metabolites exhibit excellent fungicide activity, they have the potential to become lead molecules for agricultural fungicides.

## Figures and Tables

**Table 1:** ^1^H (600 MHz) NMR Data of Compounds **1–4** in *d*_6_-DMSO ^a^.

Position	*δ*_H_, Mult (*J* in Hz)			
	1	2	3	4
2 α	2.74, d (15.6)	2.57, q (7.8)	2.76, d (16.2)	2.57, q (7.8)
2 β	2.51, overlap		2.56, d (16.2)	
2-CH_3_		1.12, overlap		1.13, d (7.2)
3-OH	5.49, s	4.93, s	5.50, overlap	4.93, s
4 α	2.16, d (17.4)	2.08, m	2.18, m	2.07, m
4 β	1.92, m	2.00, m	1.97, m	2.03, m
5	5.45, m	5.46, m	5.45, m	5.46, m
6-CH_3_	1.41, s	1.41, s	1.41, s	1.41, s
7	4.33, brs	4.30, brs	4.35, brs	4.30, brs
8-CH_3_	1.37, s	1.36, s	1.37, s	1.36, s
9	5.38, m	5.24, t (7.2)	5.50, overlap	5.41, dd (10.2, 4.2)
10 α	2.01, m	2.08, m	2.11, m	2.07, m
10 β	1.92, m	1.79, m	1.79, m	1.79, m
11 α	1.56, m	1.27, m	1.31, m	1.46, m
11 β	1.23, m	1.12, overlap	1.23, m	1.22, m
12 α	1.47, m	1.56, m	1.53, m	1.56, m
12 β	1.23, m	1.27, m	1.44, m	1.32, m
13	3.78, m	3.89, dd (12.6, 7.2)	4.02, m	3.92, m
14	5.30, dd (15.6, 8.4)	5.40, dd (10.2, 4.2)	5.28 m	5.25, m
15	5.15, dd (15.6, 9.0)	5.24, t (7.2)	5.40, m	5.25, m
16 α	1.92, m	2.00, m	2.04, m	2.07, m
16 β			1.97, m	
16-CH_3_	0.93, d (6.6)	0.90, overlap		0.91, d (6.6)
17 α	1.29, m	1.27, m	1.31, m	1.22, m
17 β	0.90, overlap	0.90, overlap	0.89, overlap	0.89, overlap
18 α	1.47, m	1.69, m	1.74, m	1.70, m
18 β	1.29, m			
18-CH_3_		0.77, d (6.6)	0.82, d (7.2)	0.77, d (6.6)
19	4.72, m	4.54, dd (6.6, 4.8)	4.64, m	4.54, m
20	1.71, m	1.79, m	1.69, m	1.79, m
20-CH_3_	0.80, d (6.6)	0.79, d (6.6)	0.81, d (7.2)	0.79, d (6.6)
21 α	1.29, m	1.27, m	1.23, m	1.32, m
21 β	0.98, m	0.95, m	0.84, d (7.2)	0.95, m
22	1.56, m	1.56, m	1.53, m	1.56, m
22-CH_3_	0.71, d (6.6)	0.72, d (6.6)	0.69, d (6.6)	0.72, d (6.6)
23	3.42, m	3.40, m	3.41, m	3.40, m
23-OH	4.88, brs		5.05, brs	5.04, brs
24	2.62, m	2.62, m	2.61, m	2.62, m
24-CH_3_	0.85, d (6.6)	0.85, d (7.2)	0.84, d (7.2)	0.85, d (7.2)
26 α	2.51, overlap	2.53, m	2.49, m	2.53, m
26 β	2.01, m	2.00, m	1.97, m	2.03, m
27	0.90, overlap	0.90, overlap	0.89, overlap	0.89, overlap
1′	4.41, dd (9.6, 1.8)	4.47, dd (9.6, 1.8)	4.58, m	4.54, m
2′ α	1.92, m	1.89, m	2.04, m	2.03, m
2′ β	1.29, m	1.27, m	1.31, m	1.22, m
3′	4.47, m	3.40, m	4.49, m	4.47, m
3′-OH	4.88, brs			
3′-CONH_2_			6.44, brs	6.48, brs
4′	2.68, m	2.68, m	2.91, m	2.91, m
4′-OH				4.56, brs
5′	3.01, m	3.03, m	3.19, m	3.14, m
5′-CH_3_	1.10, d (6.0)	1.12, overlap	1.15, d (6.0)	1.15, d (6.0)

^a^ Assignments were based on COSY, HSQC and gHMBC experiments. NMR: nuclear magnetic resonance); DMSO: dimethyl sulfoxide; COSY: correlation spectroscopy; HSQC. heteronuclear singular quantum correlation); gHMBC: gradient heteronuclear multiple bond correlation.

**Table 2:** ^13^C (150 MHz) NMR Data of Compounds **1**–**4** in *d*_6_-DMSO.

Position	*δ*_C_, Type			
	1	2	3	4
1	171.7, s	176.0, s	171.9, s	176.0, s
2	44.0, t	47.6, d	43.6, t	47.6, d
2-CH_3_		13.0, q		13.0, q
3	93.5, s	95.7, s	93.4, s	95.7, s
4	34.4, t	32.7, t	34.6, t	32.7, t
5	117.5, d	117.5, d	117.5, d	117.4, d
6	131.7, s	131.9, s	131.8, s	131.9, s
6-CH_3_	18.8, q	18.9, q	18.9, q	18.9, q
7	79.2, d	79.0, d	79.1, d	79.0, d
8	134.3, s	133.5, s	134.0, s	133.6, s
8-CH_3_	11.1, q	10.7, q	10.8, q	10.7, q
9	129.3, d	129.7, d	129.1, d	129.0, d
10	26.7, t	27.2, t	26.7, t	27.1, t
11	25.3, t	25.7, t	24.7, t	25.7, t
12	29.0, t	33.9, t	32.9, t	33.8, t
13	81.3, d	80.0, d	78.4, d	80.2, d
14	131.8, d	129.0, d	131.1, d	129.6, d
15	135.7, d	138.3, d	137.4, d	138.5, d
16	37.2, d	36.4, d	30.4, t	36.4, d
16-CH_3_	21.1, q	21.6, q		21.6, q
17	32.8, t	40.4, t	34.3, t	40.4, t
18	29.0, t	32.7, d	33.9, d	32.7, d
18-CH_3_		16.5, q	12.6, q	16.5, q
19	78.3, d	82.2, d	80.9, d	82.2, d
20	31.9, d	31.2, d	31.5, d	31.2, d
20-CH_3_	16.0, q	16.2, q	15.5, q	16.2, q
21	34.6, t	35.0, t	36.8, t	35.0, t
22	31.2, d	31.3, d	31.3, d	31.3, d
22-CH_3_	11.1, q	11.0, q	10.9, q	10.9, q
23	76.0, d	76.3, d	76.3, d	76.3, d
24	49.0, d	49.1, d	48.9, d	49.1, d
24-CH_3_	13.4, q	13.4, q	13.4, q	13.4, q
25	214.4, s	214.4, s	214.5, s	214.4, s
26	35.2, t	35.2, t	35.3, t	35.2, t
27	7.4, q	7.4, q	7.4, q	7.4, q
1′	98.7, d	97.5, d	96.7, d	97.0, d
2′	40.0, t	40.0, t	37.5, t	37.5, t
3′	70.5, d	70.5, d	72.6, d	72.7, d
3′-CONH_2_			156.4, s	156.4, s
4′	76.9, d	76.9, d	73.5, d	73.5, d
5′	71.5, d	71.5, d	71.7, d	71.7, d
5′-CH_3_	18.1, q	18.1, q	18.0, q	18.0, q

**Table 3:** Half maximal effective concentration (EC_50_) values of Compounds **1–6** on *P. oryzae* ^a^.

Compound	Mycelial Growth Inhibition EC_50_, µg/mL (±SD)	Conidial Germination Inhibition EC_50_, µg/mL (±SD)
venturicidin G, **1**	1.78 ± 0.09	24.95 ± 1.63
venturicidin H, **2**	1.43 ± 0.02	5.55 ± 0.12
venturicidin I, **3**	0.35 ± 0.03	1.14 ± 0.03
venturicidin J, **4**	1.40 ± 0.09	4.49 ± 0.28
venturicidin A, **5**	0.11 ± 0.00	0.27 ± 0.02
venturicidin B, **6**	0.15 ± 0.01	0.39 ± 0.01
Carbendazim ^b^	0.30 ± 0.01	3.99 ± 0.08

^a^ Data shown are the mean of three independent experiments and presented as mean ± standard deviation (SD). ^b^ Positive control.

## Data Availability

Not applicable.

## Supplementary Materials

The following supporting information can be downloaded at: https://www.mdpi.com/article/10.3390/microorganisms10081612/s1, Table S1: ^1^H (600 MHz) and ^13^C (150 MHz) NMR Data of Compounds **5** and **6** in *d*_6_-DMSO; Figure S1: Effects of crude extract of *Streptomyces* sp. SN5452 on the mycelial growth of *P. oryzae*; Figure S2: Neighbour-joining phylogenetic tree based on the 16S rRNA gene sequences with members of the genus Streptomyces; Figures S3–S28: HRESI-MS and NMR spectra of compounds **1–4**.
