# Supplementary material for: Macrolides from Streptomyces sp. SN5452 and Their Antifungal Activity against Pyricularia oryzae
Source: Microorganisms. 2022 Aug 9;10(8):1612. doi: 10.3390/microorganisms10081612 (PMC9416504; doi:10.3390/microorganisms10081612)
Supplement: Supplementary file 1 [file microorganisms-10-01612-s001.zip › microorganisms-1858881-supplementary for pub.pdf]

Supporting information

Macrolides from *Streptomyces* sp. SN5452 and Their Antifungal Activity against *Pyricularia oryzae*

Yinan Wang [1], Di Yang [1], Yuhui Bi [1] and Zhiguo Yu [1,2,*]

[1] College of Plant Protection, Shenyang Agricultural University, Shenyang 110866, China; yinan7765@163.com (Y.W.); yd_yangdi@163.com (D.Y.); biyuhui@syau.edu.cn (Y.B.)

[2] Engineering & Technological Research Center of Biopesticide for Liaoning Province, Shenyang 110866, China

* Correspondence: zyu@syau.edu.cn; Tel.: +86-24-88487148

Contents

Table S1.^{1}H (600 MHz) and ^{13}C (150 MHz) NMR Data of Compounds **5** and **6** in *d*$_6$-DMSO 1

Figure S1. Effects of crude extract of *Streptomyces* sp. SN5452 on the mycelial growth of *P. oryzae*. 2

Figure S2. Neighbour-joining phylogenetic tree based on the 16S rRNA gene sequences with members of the genus *Streptomyces* 3

Figure S3. The ^{1}H-NMR spectrum of Venturicidin G (**1**) in *d*$_6$-DMSO. 4

Figure S4. The ^{13}C-NMR spectrum of Venturicidin G (**1**) in *d*$_6$-DMSO. 4

Figure S5. The HSQC spectrum of Venturicidin G (**1**) in *d*$_6$-DMSO 5

Figure S6. The HMBC spectrum of Venturicidin G (**1**) in *d*$_6$-DMSO 5

Figure S7. The ^{1}H-^{1}H COSY spectrum of Venturicidin G (**1**) in *d*$_6$-DMSO 6

Figure S8. The HRESIMS spectrum of Venturicidin G (**1**) 6

Figure S9. The ^{1}H-NMR spectrum of Venturicidin H (**2**) in *d*$_6$-DMSO 7

Figure S10. The ^{13}C-NMR spectrum of Venturicidin H (**2**) in *d*$_6$-DMSO 7

Figure S11. The HSQC spectrum of Venturicidin H (**2**) in *d*$_6$-DMSO 8

Figure S12. The HMBC spectrum of Venturicidin H (**2**) in *d*$_6$-DMSO 8

Figure S13. The ^{1}H-^{1}H COSY spectrum of Venturicidin H (**2**) in *d*$_6$-DMSO 9

Figure S14. The NOESY spectrum of Venturicidin H (**2**) in *d*$_6$-DMSO 9

Figure S15. The HRESIMS spectrum of Venturicidin H (**2**) 10

Figure S16. The ^{1}H-NMR spectrum of Venturicidin I (**3**) in d_6-DMSO 10

Figure S17. The ^{13}C-NMR spectrum of Venturicidin I (**3**) in d_6-DMSO 11

Figure S18. The HSQC spectrum of Venturicidin I (**3**) in d_6-DMSO 11

Figure S19. The HMBC spectrum of Venturicidin I (**3**) in d_6-DMSO 12

Figure S20. The ^{1}H-^{1}H COSY spectrum of Venturicidin I (**3**) in d_6-DMSO 12

Figure S21. The HRESIMS spectrum of Venturicidin I (**3**) 13

Figure S22. The ^{1}H-NMR spectrum of Venturicidin J (**4**) in d_6-DMSO 13

Figure S23. The ^{13}C-NMR spectrum of Venturicidin J (**4**) in d_6-DMSO 14

Figure S24. The HSQC spectrum of Venturicidin J (**4**) in d_6-DMSO 14

Figure S25. The HMBC spectrum of Venturicidin J (**4**) in d_6-DMSO 15

Figure S26. The ^{1}H-^{1}H COSY spectrum of Venturicidin J (**4**) in d_6-DMSO 15

Figure S27. The NOESY spectrum of Venturicidin J (**4**) in d_6-DMSO 16

Figure S28. The HRESIMS spectrum of Venturicidin J (**4**) 16

Table S1. ^{1}H (600 MHz) and ^{13}C (150 MHz) NMR Data of compounds **5** and **6** in d_6-DMSO

Position	δ_H, type		δ_C, type	
	**5**	**6**	**5**	**6**
1			171.9, s	171.9, s
2 α	2.73, d (16.2)	2.73, d (15.6)	43.6, t	43.7, t
2 β	2.55, d (16.2)	2.54, m		
2-CH_3				
3			93.4, s	93.4, s
3-OH	5.47, s	5.47, s		
4 α	2.16, d (16.8)	2.16, d (16.2)	34.8, t	31.1, t
4 β	1.99, d (12.6)	1.98, m		
5	5.44, m	5.45, m	117.4, d	117.5, d
6			131.8, s	131.8, s
6-CH_3	1.41, s	1.41, s	18.9, q	19.5, q
7	4.32, s	4.32, s	79.2, d	79.2, d
8			134.1, s	134.1, s
8-CH_3	1.36, s	1.36, s	10.8, q	10.8, q
9	5.41, m	5.41, dd (10.2, 4.2)	129.1, d	130.0, d
10 α	2.06, m	2.07, m	26.8, t	26.9, t
10 β	1.86, m	1.85, m		
11 α	1.36, s	1.27, m	25.5, t	25.5, t
11 β	1.14, d (6.0)	1.11 d (6.0)		
12 α	1.55, m	1.54, m	34.2, t	34.3, t
12 β	1.33, s	1.33, m		
13	3.86, m	3.83, m	80.8, d	82.3, d
14	5.29, m	5.29, dd (15.6, 8.4)	129.8, d	129.1, d
15	5.23, m	5.21, dd (15.6, 9.0)	137.4, d	137.2, d
16 α	2.06, m	2.07, m	34.6, d	34.7, d
16 β				
16-CH_3	0.89, m	0.89, m	19.4, q	18.9, q
17 α	1.14, d (6.0)	1.27, m	41.3, t	41.3, t
17 β	0.89, m	0.96, m		
18 α	1.80, m	1.80, m	31.2, d	34.6, d
18 β				
18-CH_3	0.78, d (6.6)	0.78, d (7.2)	12.8, q	12.9, q
19	4.58, d (6.6)	4.55, dd (7.8, 4.2)	82.3, d	80.5, d
20	1.74, m	1.79, m	31.5, d	31.2, d
20-CH_3	0.81, d (6.6)	0.81, d (6.6)	15.8, q	15.8, q

Position	δH, type		δC, type	
	**5**	**6**	**5**	**6**
21 α	1.30, m	1.27, m	36.2, t	36.2, t
21 β	0.84, d (7.2)	0.84, d (7.2)		
22	1.55, m	1.73, m	31.1, d	31.5, d
22-CH_3	0.70, d (6.6)	0.70, d (6.6)	10.9, q	10.9, q
23	3.36, m	3.36, m	76.3, d	76.9, d
23-OH	4.54, m			
24	2.62, m	2.62, m	48.9, d	49.0, d
24-CH_3	0.84, d (7.2)	0.84, d (7.2)	13.4, q	13.4, q
25			214.4, s	214.5, s
26 α	2.62, m	2.68, t (9.0)	35.4, t	35.4, t
26 β	2.47, m	2.49, m		
27	0.89, m	0.89, m	7.4, q	7.4, q
1′	4.54, m	4.45, dd (9.6, 1.8)	97.6, d	98.1, d
2′ α	2.06, m	1.90, m	37.5, t	40.0, t
2′ β	1.30, m	1.33, m		
3′	4.47, m	3.36, m	72.7, d	70.5, d
3′-$CONH_2$	6.46, s			
3′-$CONH_2$			156.4, s	
3′-OH		4.89, s		
4′	2.91, m	3.03, m	73.5, d	76.3, d
4′-OH	5.02, d (6.0)			
5′	3.17, m	3.36, m	71.7, d	71.5, d
5′-CH_3	1.14, d (6.0)	1.11, d (6.0)	18.0, q	18.1, q

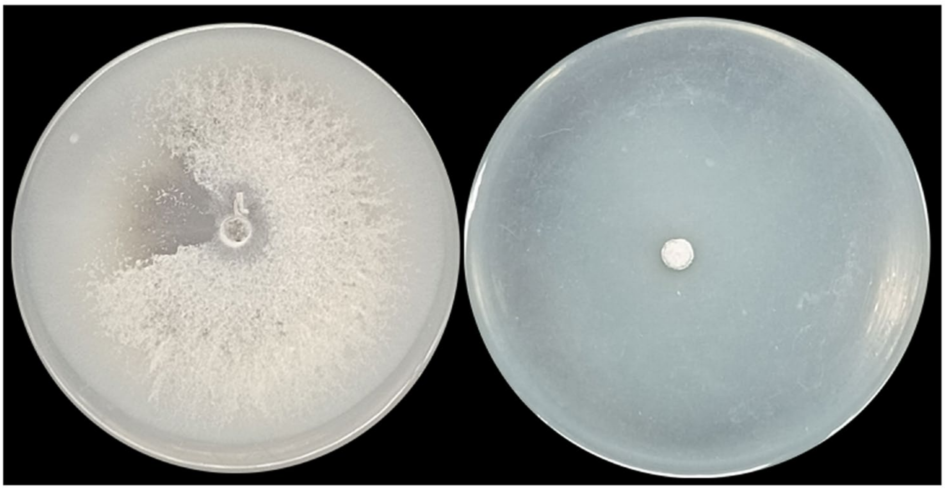

Figure S1. Effects of crude extract of *Streptomyces* sp. SN5452 on the mycelial growth of *P. oryzae*

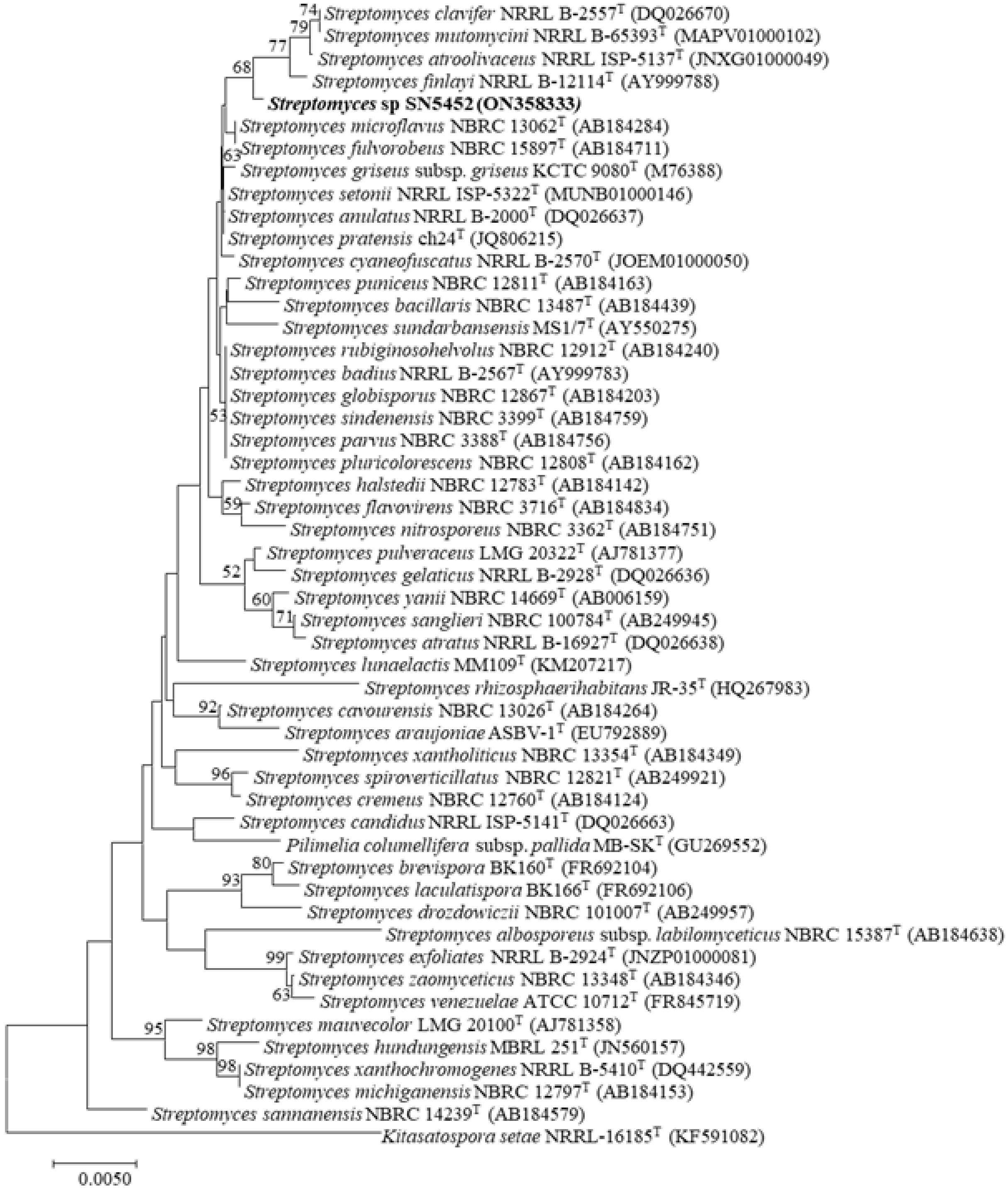

Figure S2. Neighbour-joining phylogenetic tree based on the 16S rRNA gene sequences with members of the genus *Streptomyces*. Bootstrap values (expressed as percentages of 1000 replications) of above 50 % are shown at branch points. GenBank accession numbers are given in parentheses. *Kitasatospora setae* NRRL-16185^{T} was used as the outgroup. Bar, 0.005 substitution per nucleotide position.

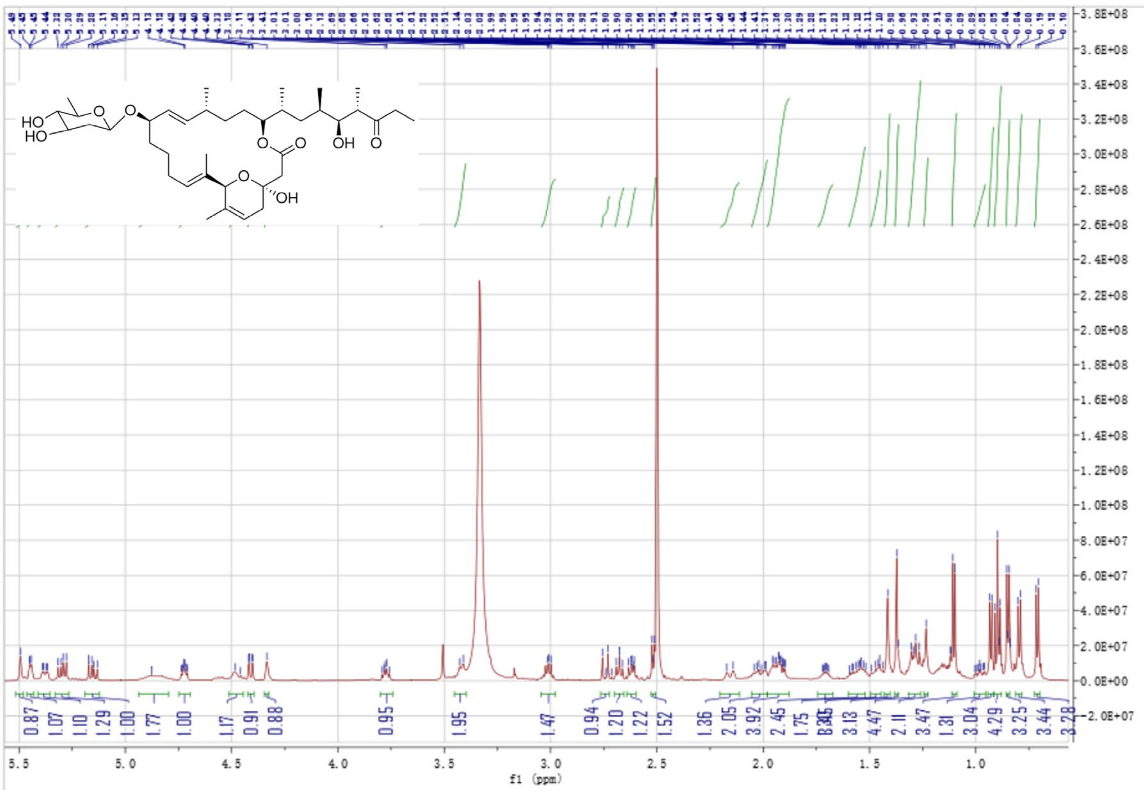

Figure S3. The ^{1}H-NMR spectrum of Venturicidin G (**1**) in *d*$_6$-DMSO

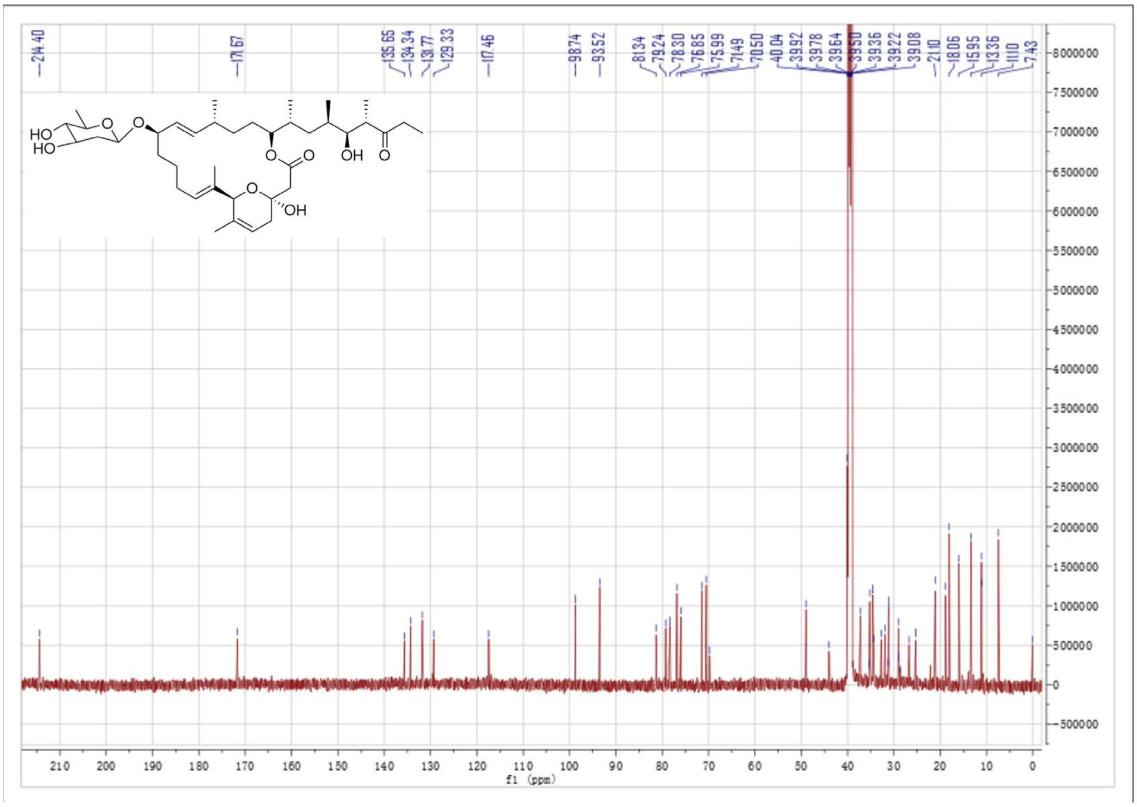

Figure S4. The ^{13}C-NMR spectrum of Venturicidin G (**1**) in *d*$_6$-DMSO

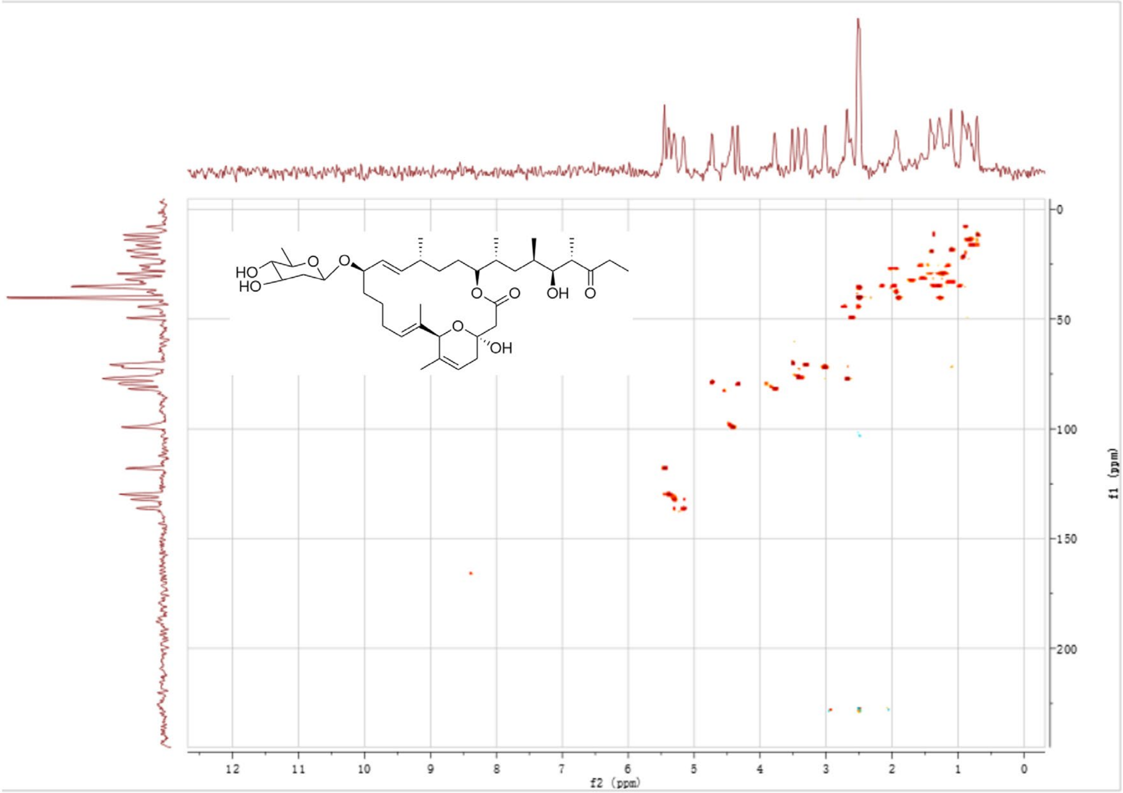

Figure S5. The HSQC spectrum of Venturicidin G (**1**) in d_6-DMSO

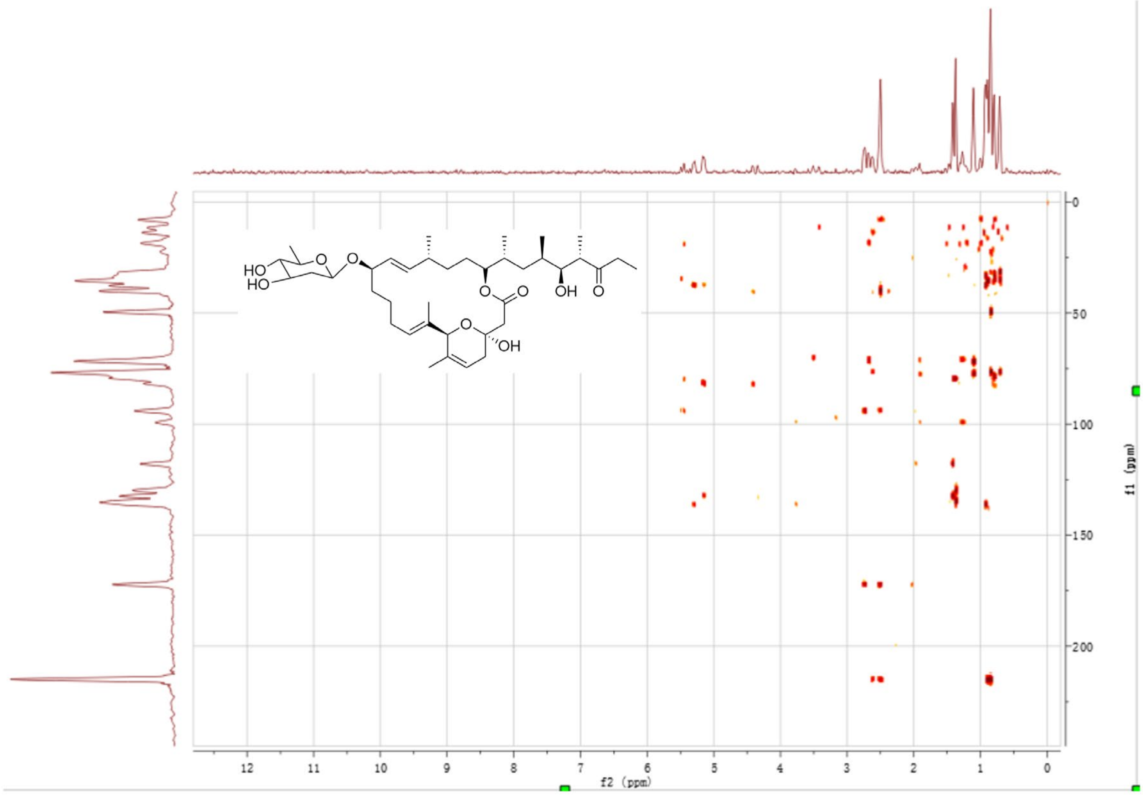

Figure S6. The HMBC spectrum of Venturicidin G (**1**) in d_6-DMSO

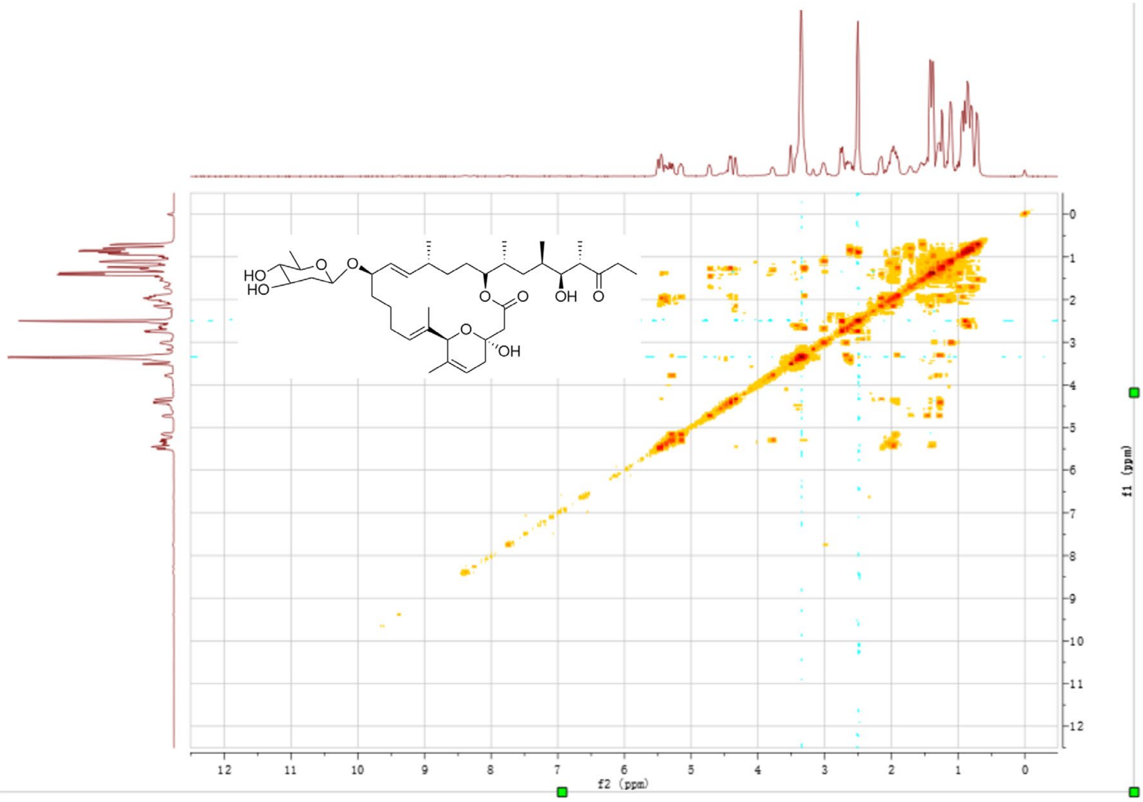

Figure S7. The ^1H-^1H COSY spectrum of Venturicidin G (**1**) in d_6-DMSO

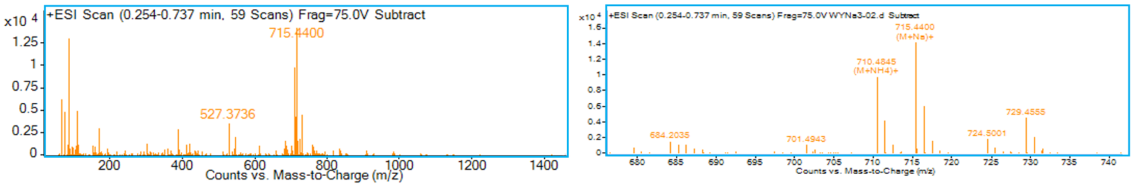

Figure S8. The HRESIMS spectrum of Venturicidin G (**1**)

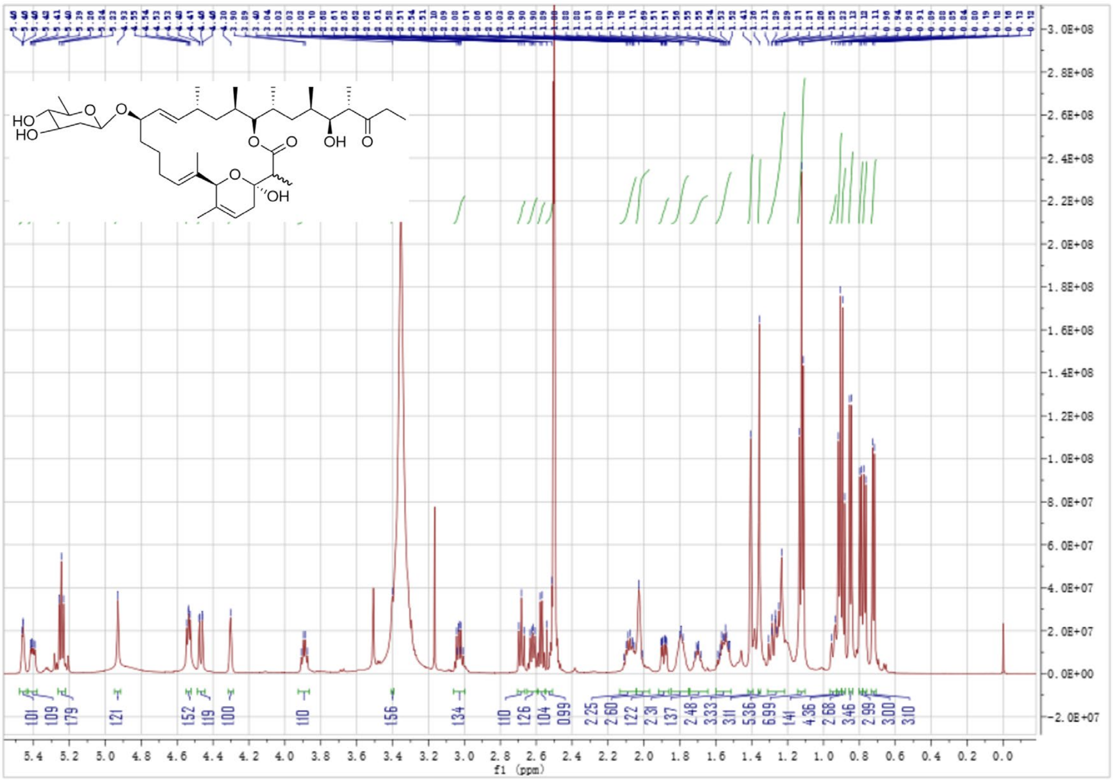

Figure S9. The ^{1}H-NMR spectrum of Venturicidin H (**2**) in *d*$_6$-DMSO

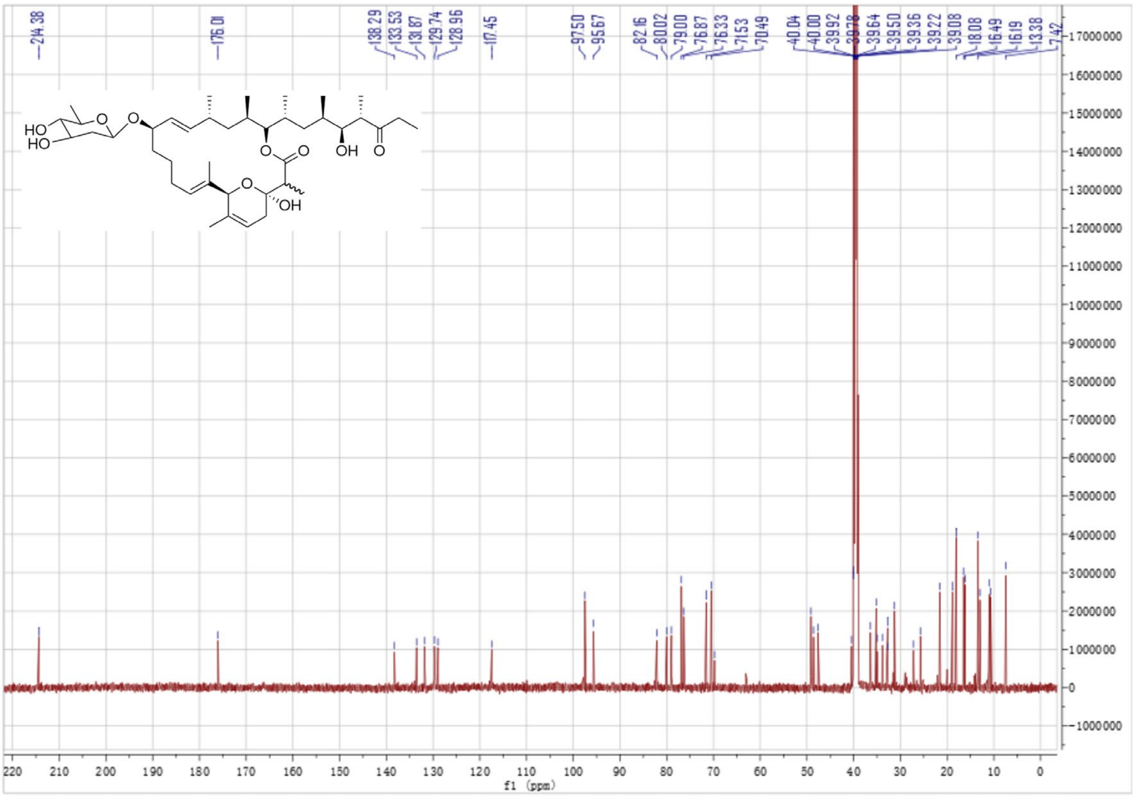

Figure S10. The ^{13}C-NMR spectrum of Venturicidin H (**2**) in *d*$_6$-DMSO

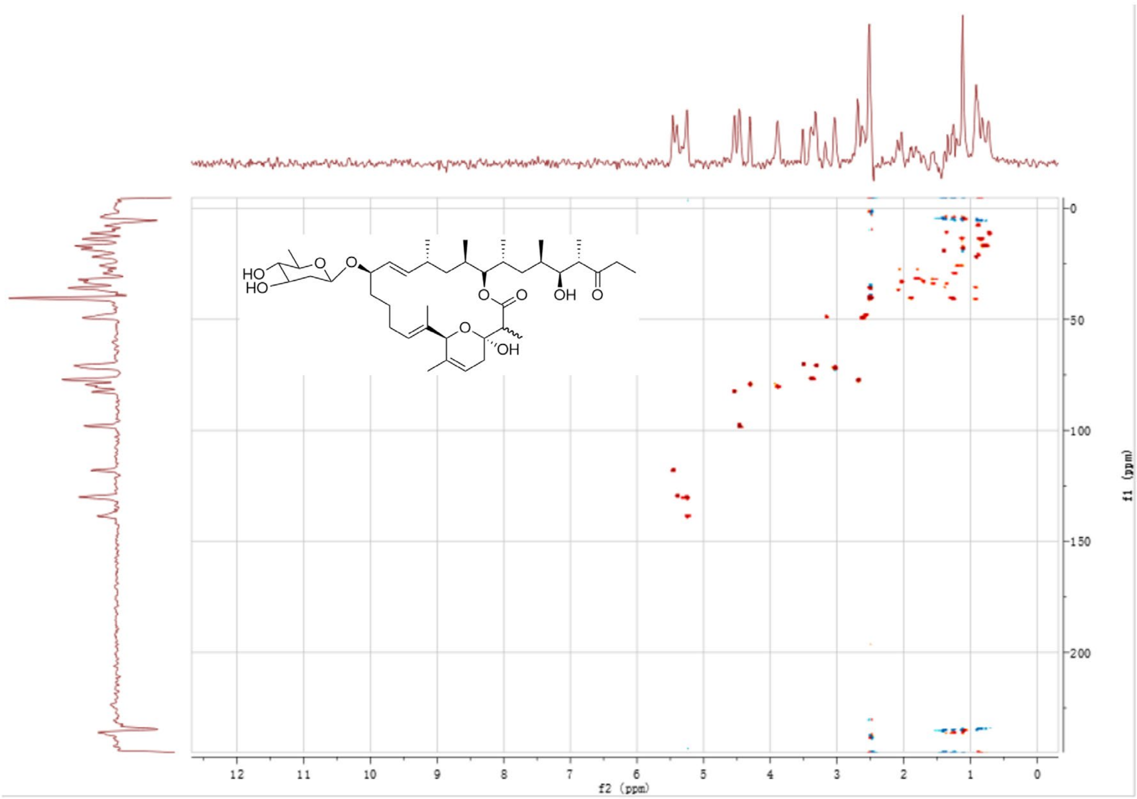

Figure S11. The HSQC spectrum of Venturicidin H (**2**) in *d*$_6$-DMSO

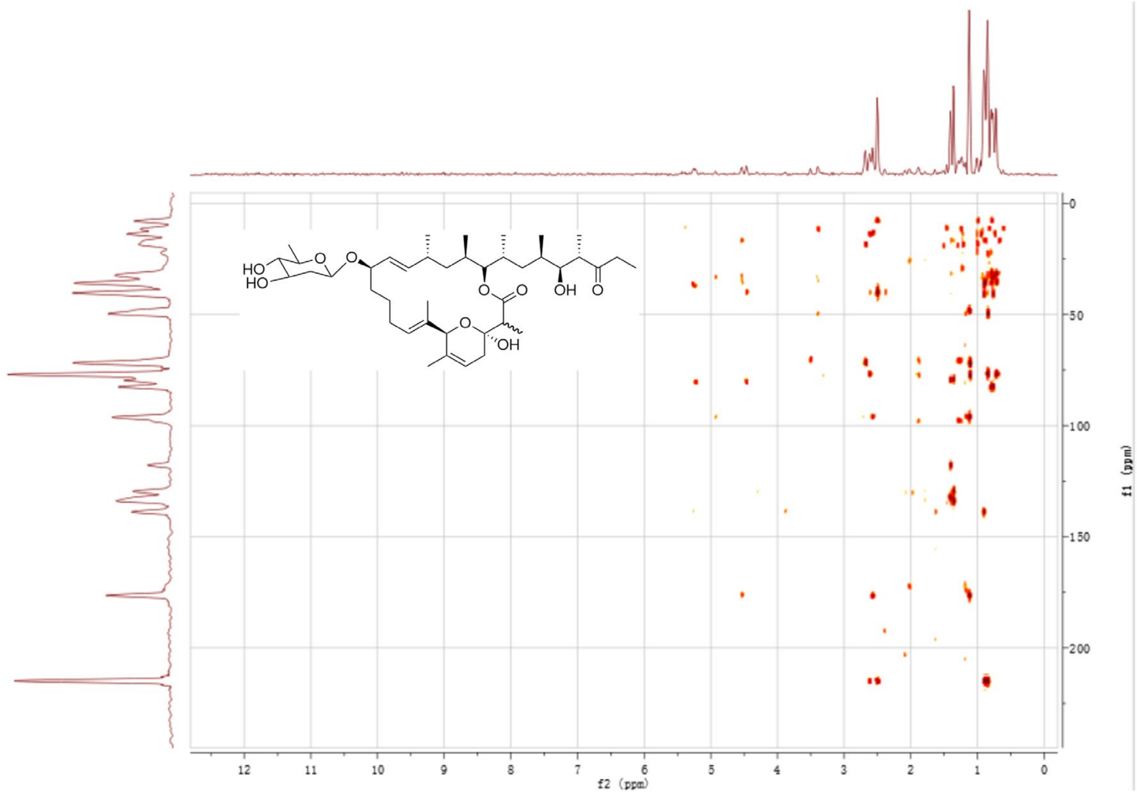

Figure S12. The HMBC spectrum of Venturicidin H (**2**) in *d*$_6$-DMSO

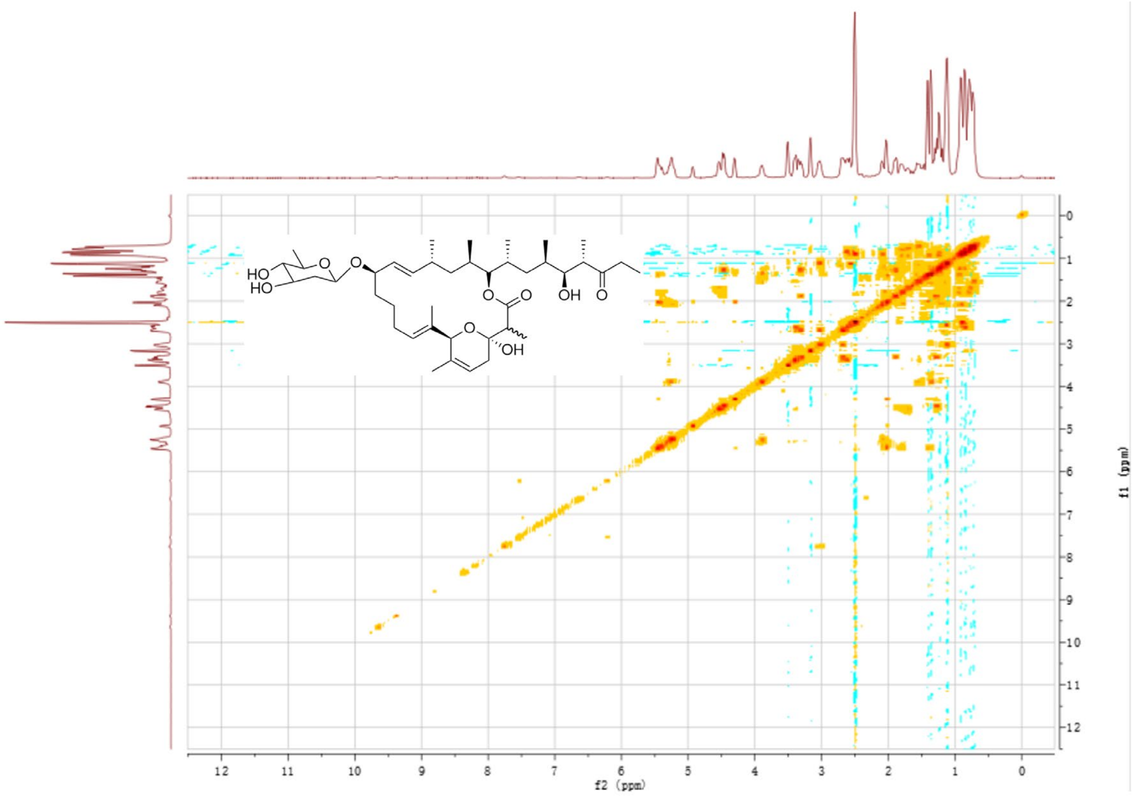

Figure S13. The ^{1}H-^{1}H COSY spectrum of Venturicidin H (**2**) in *d*$_6$-DMSO

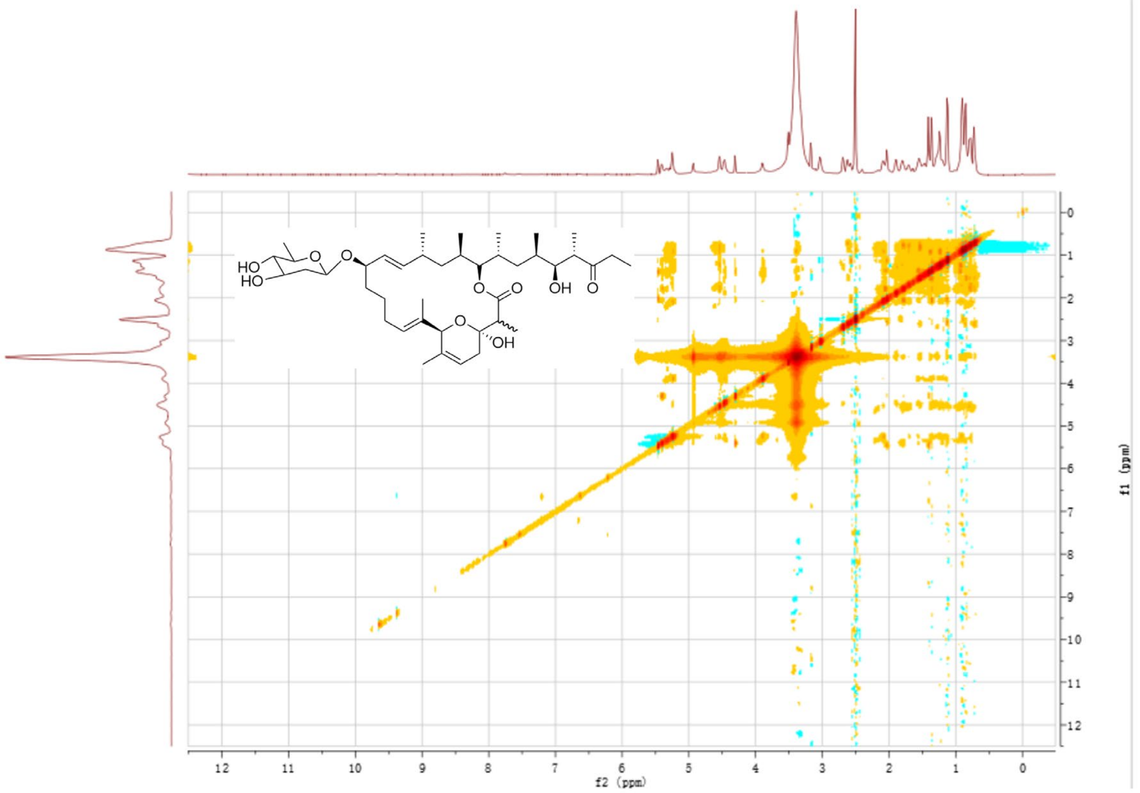

Figure S14. The NOESY spectrum of Venturicidin H (**2**) in *d*$_6$-DMSO

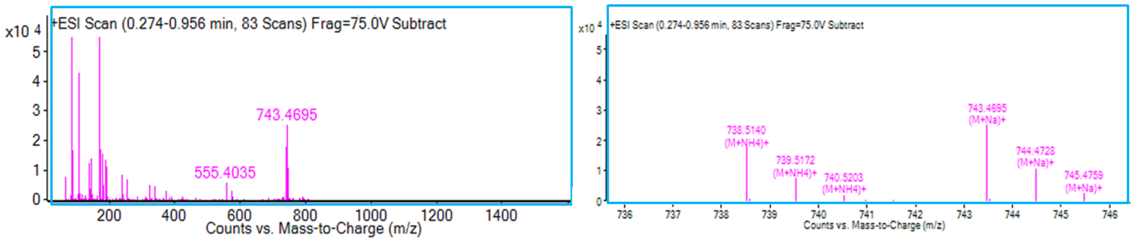

Figure S15. The HRESIMS spectrum of Venturicidin H (**2**)

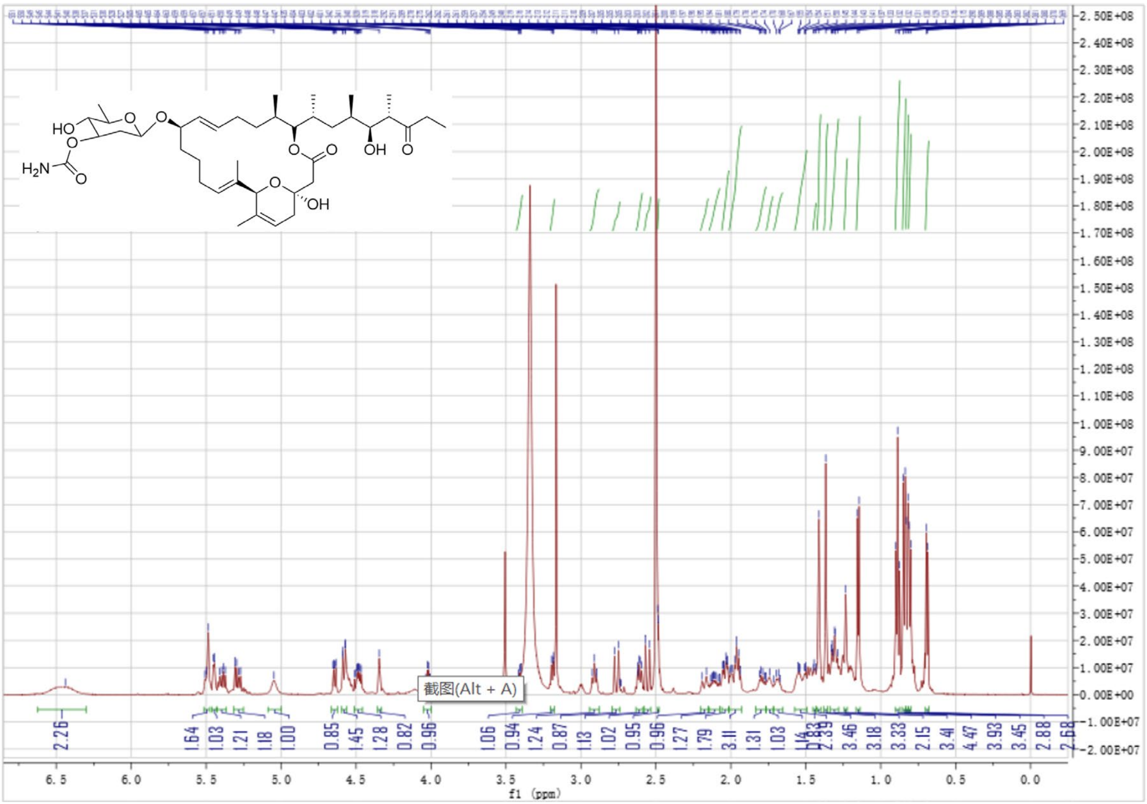

Figure S16. The ^{1}H-NMR spectrum of Venturicidin I (**3**) in d_6-DMSO

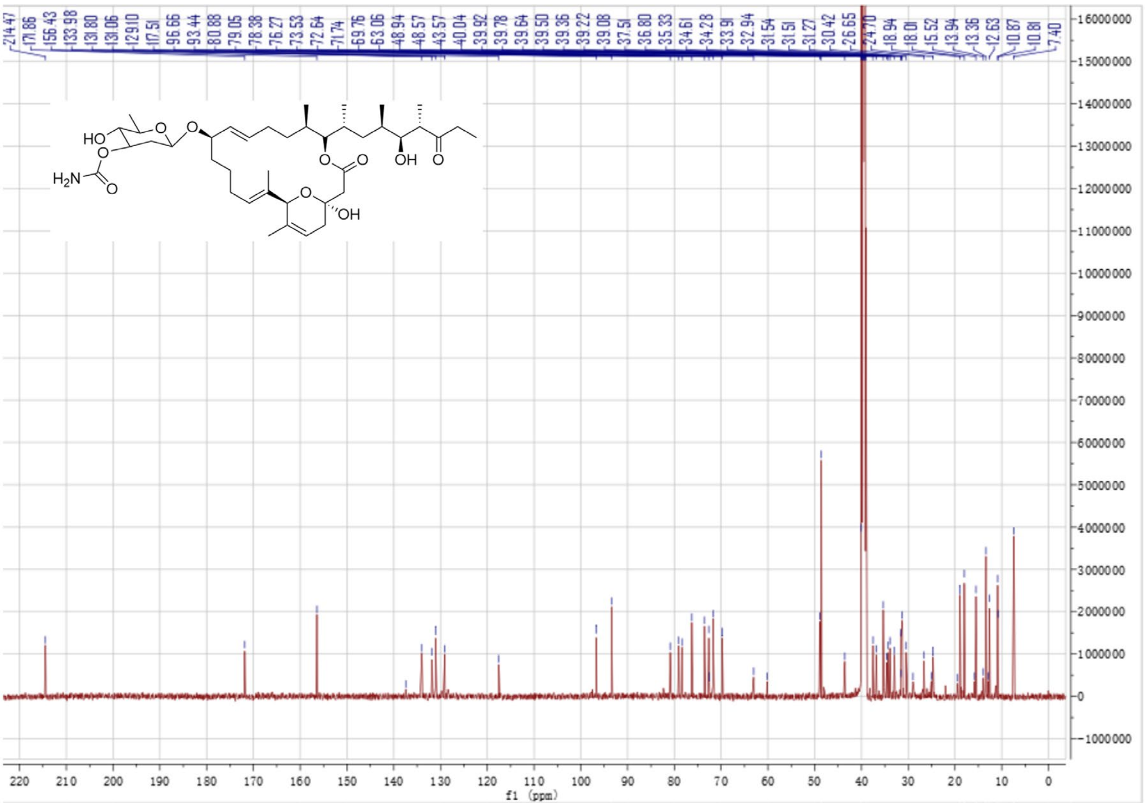

Figure S17. The ^{13}C-NMR spectrum of Venturicidin I (**3**) in *d*$_6$-DMSO

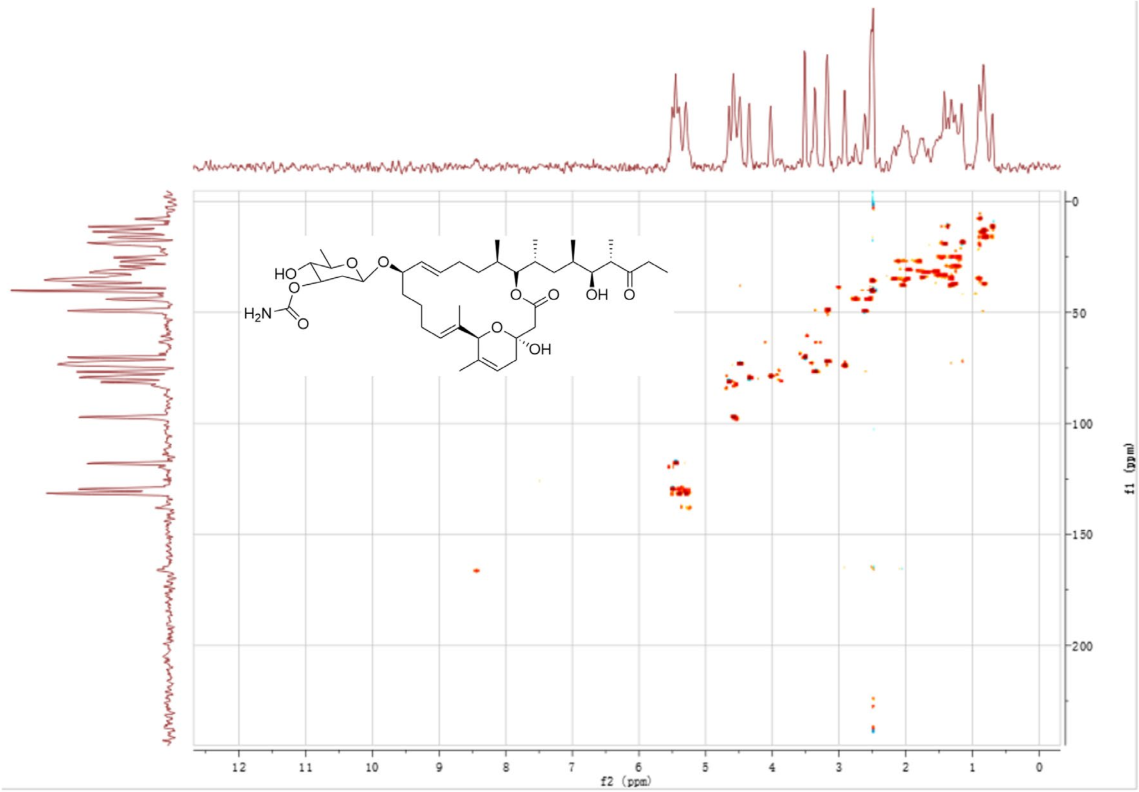

Figure S18. The HSQC spectrum of Venturicidin I (**3**) in *d*$_6$-DMSO

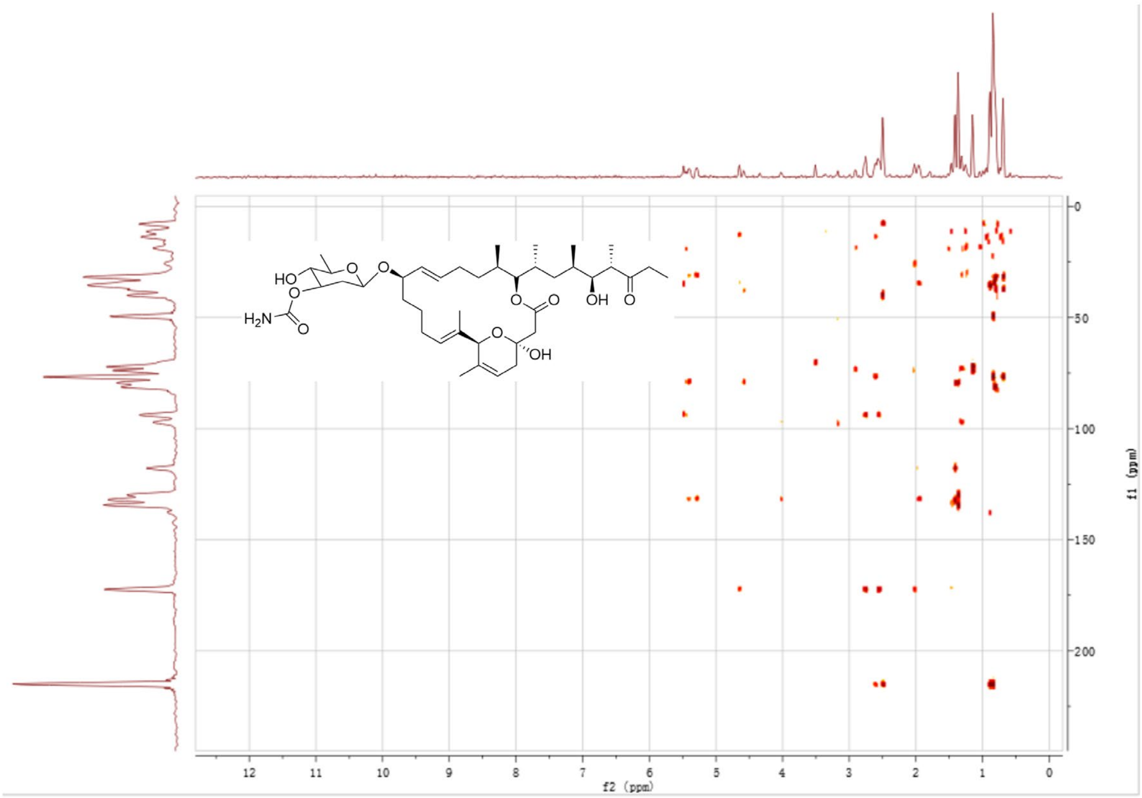

Figure S19. The HMBC spectrum of Venturicidin I (**3**) in d_6-DMSO

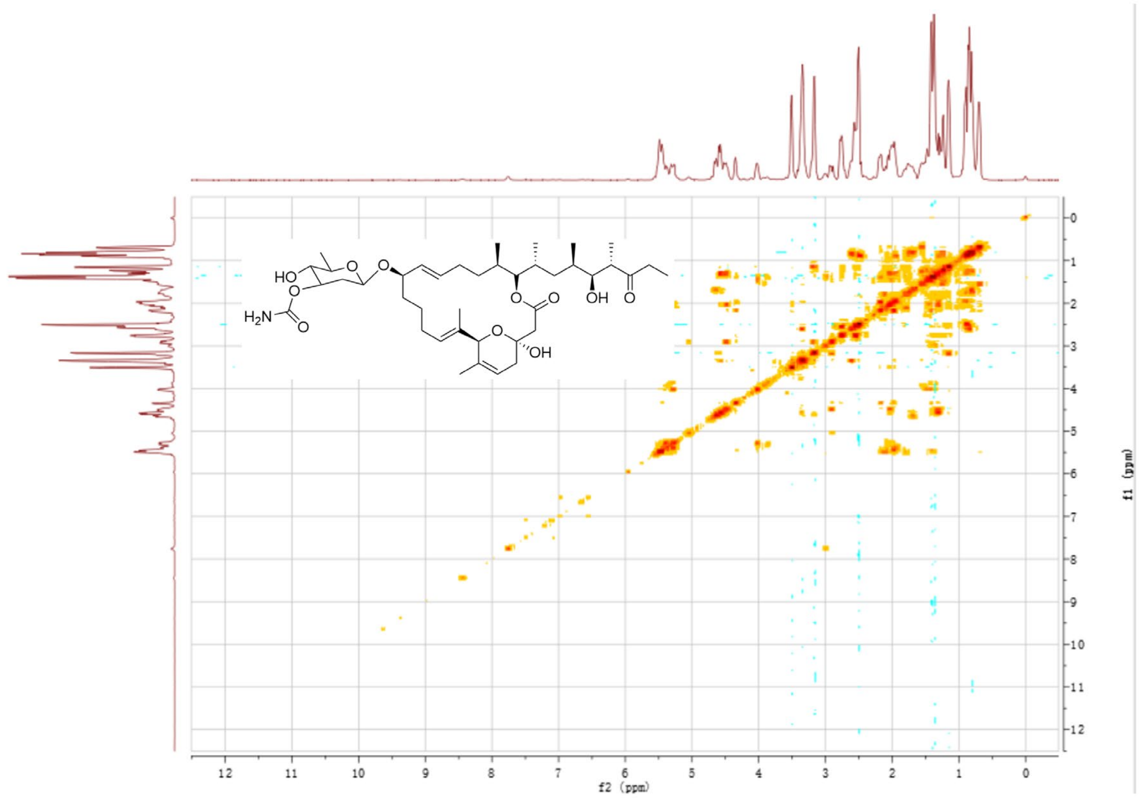

Figure S20. The ^1H-^1H COSY spectrum of Venturicidin I (**3**) in d_6-DMSO

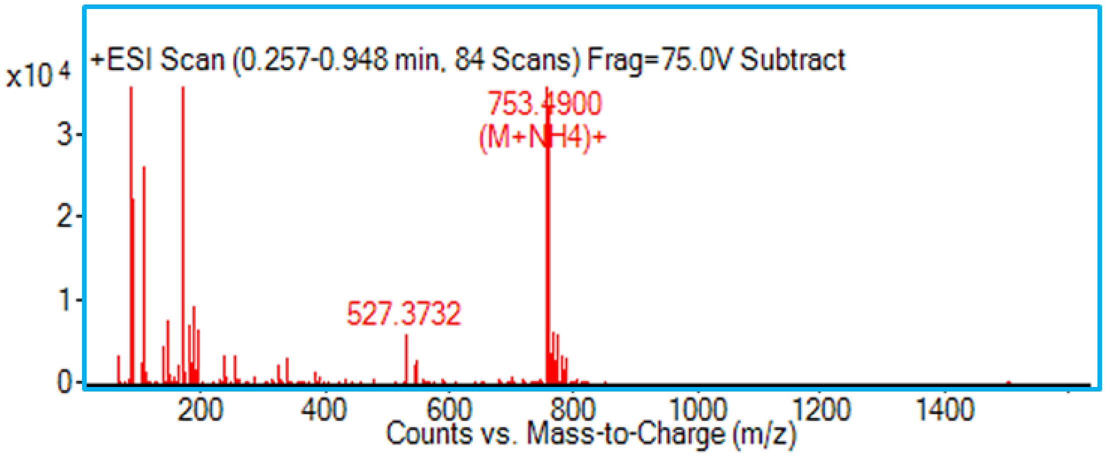

Figure S21. The HRESIMS spectrum of Venturicidin I (**3**)

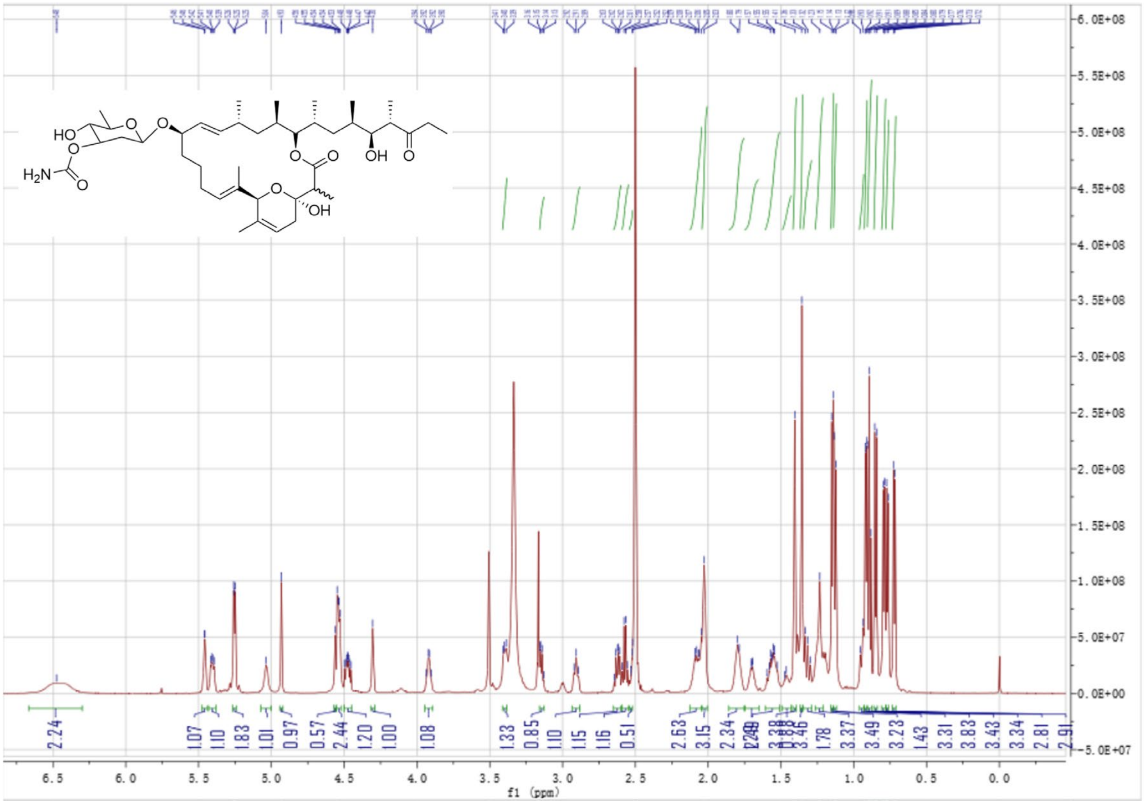

Figure S22. The ^{1}H-NMR spectrum of Venturicidin J (**4**) in d_6-DMSO

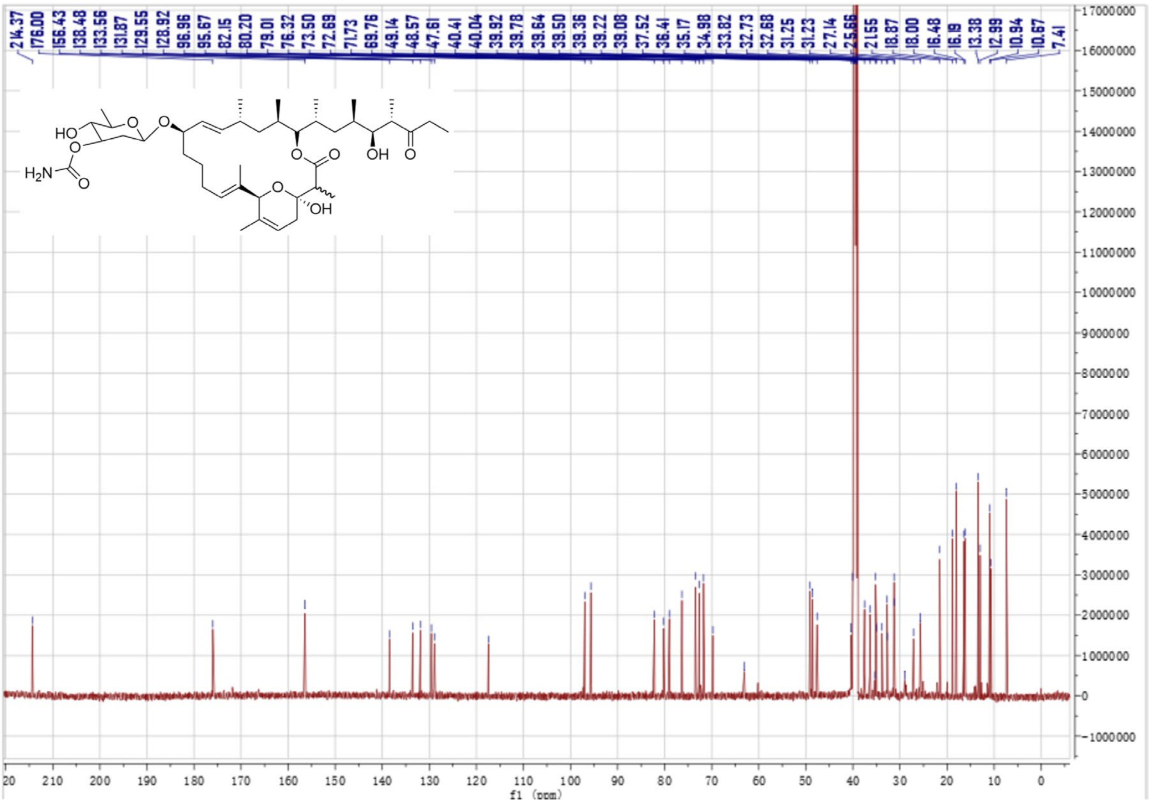

Figure S23. The ^{13}C-NMR spectrum of Venturicidin J (**4**) in *d*$_6$-DMSO

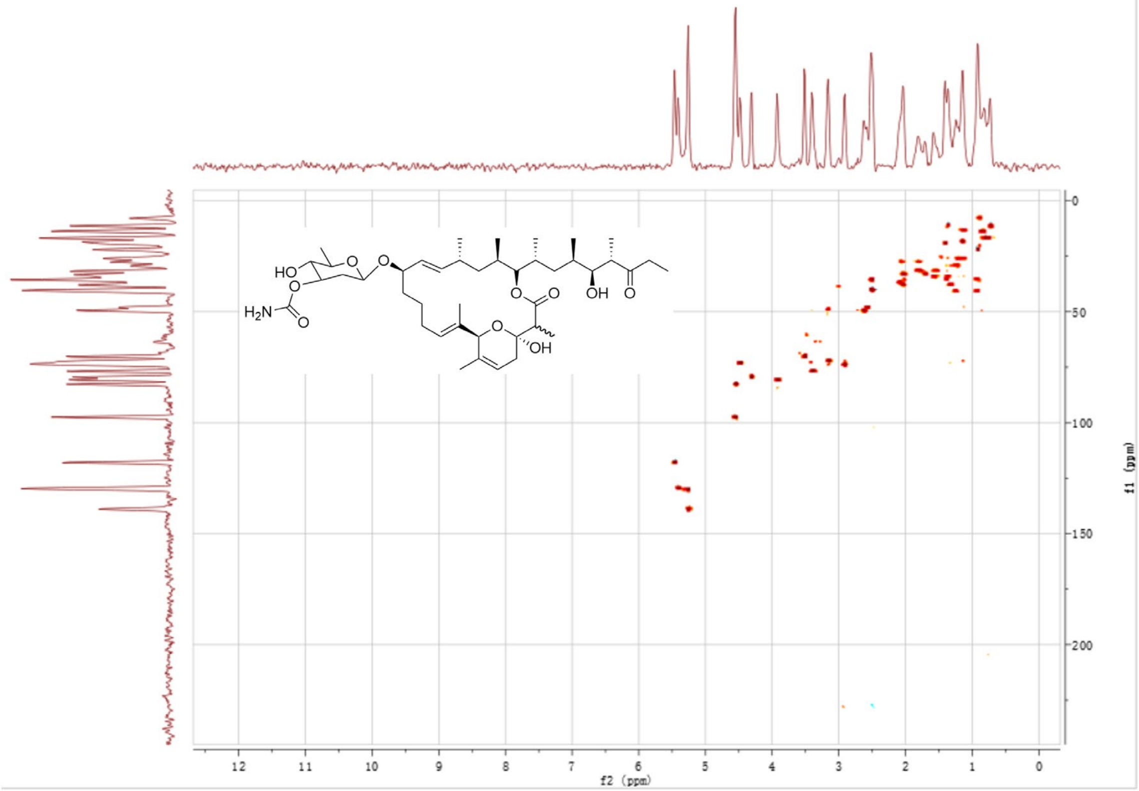

Figure S24. The HSQC spectrum of Venturicidin J (**4**) in *d*$_6$-DMSO

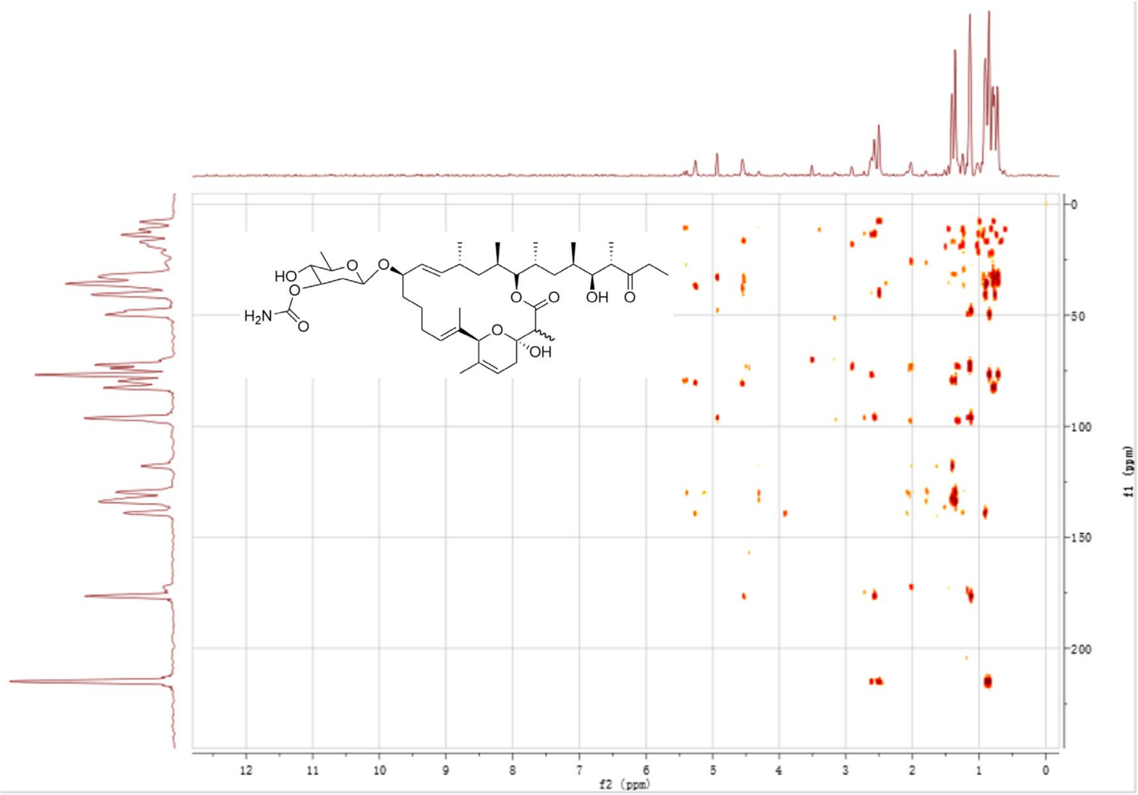

Figure S25. The HMBC spectrum of Venturicidin J (**4**) in d_6-DMSO

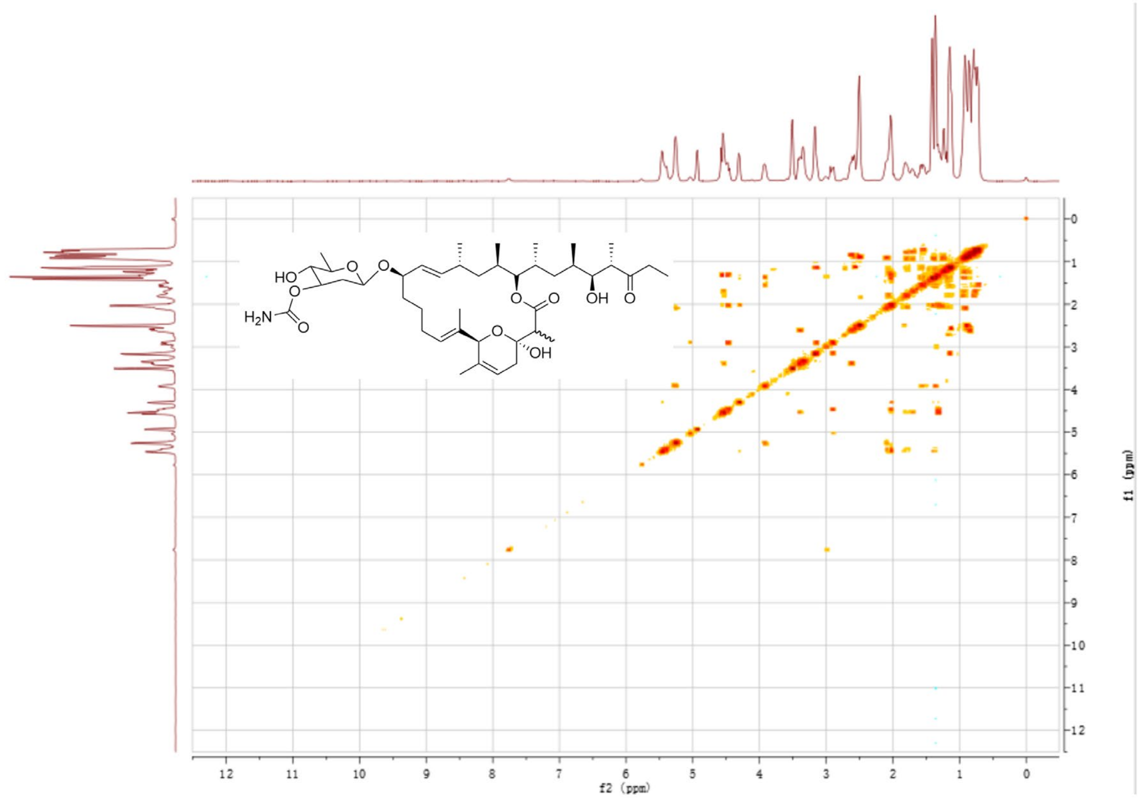

Figure S26. The ^1H-^1H COSY spectrum of Venturicidin J (**4**) in d_6-DMSO

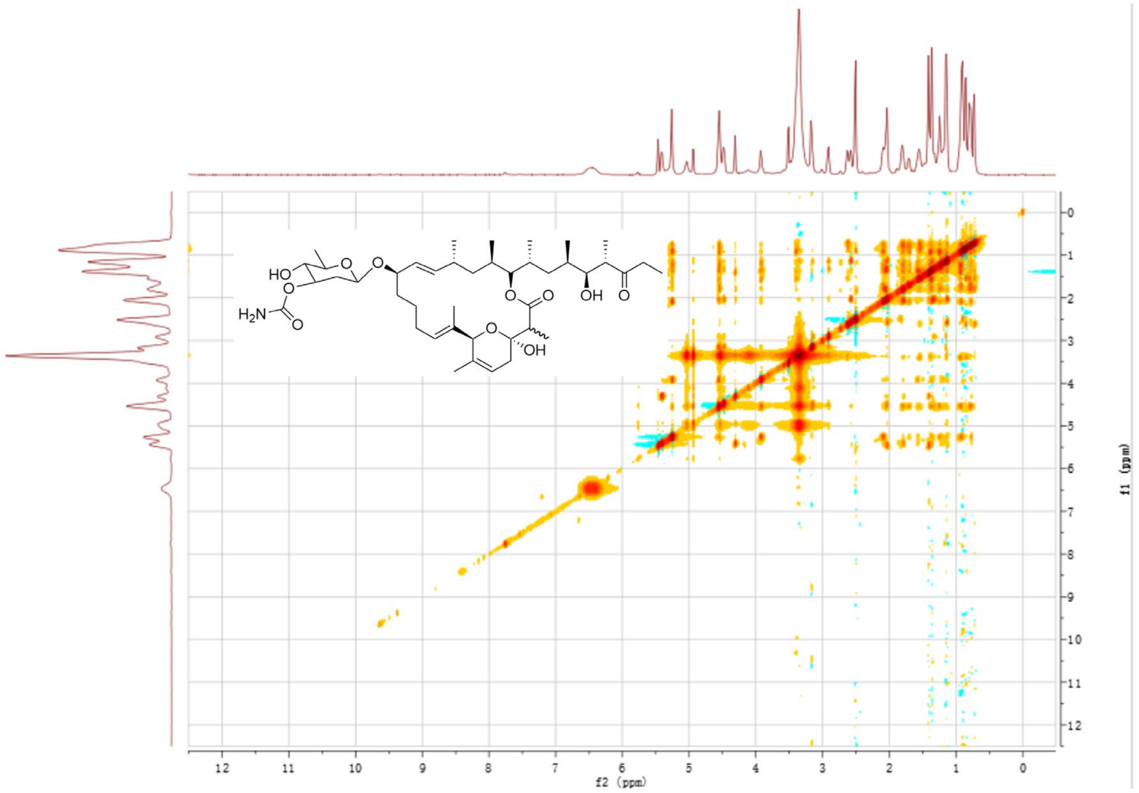

Figure S27. The NOESY spectrum of Venturicidin J (**4**) in *d*6-DMSO

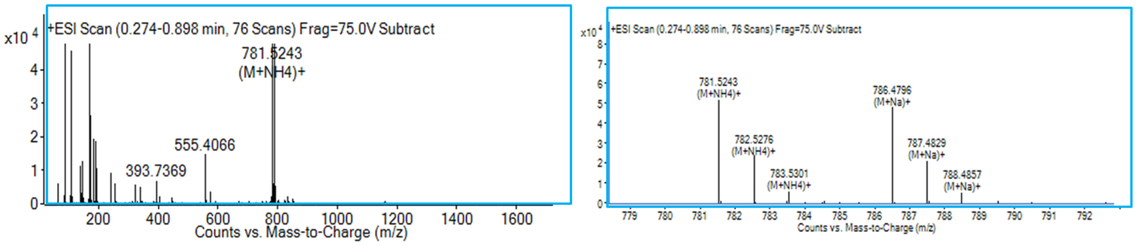

Figure S28. The HRESIMS spectrum of Venturicidin J (**4**)
